# Mechanism of age-related accumulation of mtDNA mutations in human blood

**DOI:** 10.1038/s41586-026-10569-6

**Published:** 2026-05-27

**Authors:** Rahul Gupta, Timothy J. Durham, Grant Chau, Masahiro Kanai, Md Mesbah Uddin, Wenhan Lu, M. Austin Argentieri, Konrad J. Karczewski, Daniel Howrigan, Pradeep Natarajan, Wei Zhou, Benjamin M. Neale, Vamsi K. Mootha

**Affiliations:** 1 Howard Hughes Medical Institute, Massachusetts General Hospital, Boston, MA USA; 2 Analytic and Translational Genetics Unit, Department of Medicine, Massachusetts General Hospital, Boston, MA USA; 3 Harvard Medical School, Boston, MA USA; 4 Metabolism Program, Broad Institute of MIT and Harvard, Cambridge, MA USA; 5 Program in Medical and Population Genetics, Broad Institute of MIT and Harvard, Cambridge, MA USA; 6 Department of Molecular Biology, Massachusetts General Hospital, Boston, MA USA; 7 Stanley Center for Psychiatric Research, Broad Institute of MIT and Harvard, Cambridge, MA USA; 8 Center for Computational and Integrative Biology, Massachusetts General Hospital, Boston, MA USA; 9 Heart and Vascular Institute, Mass General Brigham, Boston, MA USA; 10 Center for Genomic Medicine, Massachusetts General Hospital, Boston, MA USA; 11 Novo Nordisk Foundation Center for Genomic Mechanisms of Disease, Broad Institute of MIT and Harvard, Cambridge, MA USA; 12 Psychiatric and Neurodevelopmental Genetics Unit, Center for Genomic Medicine, Massachusetts General Hospital, Harvard Medical School, Boston, MA USA

**Keywords:** Mitochondrial genome, Genetic variation, Genome-wide association studies, Ageing, Mutation

## Abstract

Accumulation of mutant mitochondrial DNA (mtDNA) heteroplasmy is among the strongest signatures of ageing^1^. Here we investigated the underlying mechanism by calling mtDNA sequence, mtDNA abundance and mtDNA heteroplasmic variants in human blood using whole-genome sequences from approximately 750,000 individuals. We observed that mtDNA single-nucleotide variants (mtSNVs) accumulate sharply at age 60 years, occur at low levels of heteroplasmy, exhibit little evidence of positive selection and are likely to be predominantly neutral. The mutational spectrum of mtSNVs does not reflect oxidative lesions, as is commonly invoked, but is more consistent with mtDNA replication errors. To understand why mtSNVs become detectable with age, we performed a genome-wide association study for heteroplasmic mtSNV burden, identifying germline variants near *TERT*, *TCL1A* and *SMC4*, all of which have been linked to clonal haematopoiesis (CH)^2^. Rare-variant analysis also showed that high mtSNV burden is associated with mutations in numerous CH driver genes. These genetic associations persisted even after exclusion of individuals with known CH driver mutations. Our results support a model in which ‘cryptic’ mtDNA mutations initially arise randomly as replication errors but are undetectable in bulk. They then become apparent only through age-related expansion of cellular clones in blood. We propose that the high copy number and mutation rate of mtDNA make it a sensitive blood-based marker of somatic mosaicism due to CH. Our work mechanistically unifies three prominent signatures of ageing: common germline variants in *TERT*, CH and observed accrual of mtDNA mutations.

## Abstract (web summary)

Genome-wide analyses indicate that accumulation of mitochondrial DNA mutations, a hallmark of ageing, may be caused by cryptic replication errors, which act as passenger mutations that eventually become detectable with age owing to age-related somatic mosaicism, rather than oxidative damage.

## Main

Mitochondrial DNA (mtDNA) heteroplasmy arises when a cell or tissue contains a mixture of two or more different mtDNA alleles. Heteroplasmy dynamics tend to be complex, varying across generations, during development, in disease and with ageing. Historically, most studies of mtDNA heteroplasmy in humans have focused on rare, maternally transmitted disorders, which are typically driven by loss-of-function mtDNA mutations at high levels of heteroplasmy. However, there is growing evidence that low levels of mtDNA heteroplasmic variants are found in nearly all humans^3^. Using biobank-scale genomics, we previously reported that nearly everyone harbours two different classes of such variants in blood^4^. ‘Length heteroplasmies’ (insertion/deletion (indel) mutations within polypyrimidine tracts) do not accumulate with age, tend to be maternally transmitted and, once inherited, exhibit levels of heteroplasmy under nuclear genetic control. The associated nuclear DNA (nucDNA) loci tend to implicate mitochondria-localized proteins with established roles in mtDNA replication and maintenance. By contrast, heteroplasmic mtSNVs tend not to be inherited but rather seem to be somatic in origin and accumulate with age^4^.

The mechanism of this age-related accrual of heteroplasmic mtSNVs in blood is unknown. Classically, oxidative damage to mtDNA from reactive oxygen species has been invoked as a part of a ‘vicious cycle’ in which mtDNA mutations lead to further generation of reactive oxygen species and more mutations^5–7^. More recent studies of ageing brains^8^ and tumour samples^9^ have questioned the role of oxidative damage^10^ in mutation generation. Once individual mutations arise, it is unclear how they become abundant enough to detect.

Here we investigated why mtSNVs accumulate with age in blood. We report an analysis of mtDNA using a callset of approximately 750,000 individuals across the UK Biobank (UKB) and All of Us (AoU). We resolved the mutational spectrum of age-accumulating blood mtDNA and then performed a genome-wide association study (GWAS) for the burden of mtSNVs to identify mechanisms of control by rare and common germline nuclear genetic variants. Unexpectedly, the loci we identified were related not to mitochondrial homeostasis but rather to CH. Our analyses support a model in which individual blood cells randomly accumulate low levels of neutral mtDNA variation (that is, cryptic mutations), probably owing to replication-related errors, that are not detectable in bulk. However, with age-related expansion of individual cellular clones (by means of CH), these low-level cryptic variants become detectable and give rise to the observed accumulation with age.

## mtDNA sequences from 736,038 individuals

**Extended Data Fig. 1:**
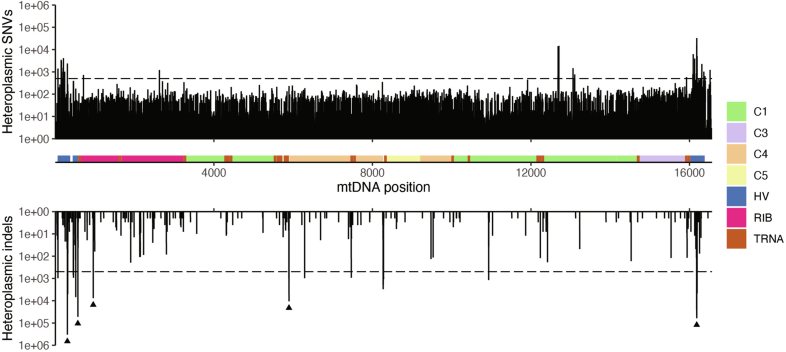
Landscape of mtDNA insertion/deletion and SNV mutations across UKB and AoU. Upper plot corresponds to SNVs, lower plot corresponds to indels, and middle scheme shows genomic annotations. C1-C5 correspond to OXPHOS genes, HV are the non-coding hypervariable regions, RIB are the ribosomal RNA genes, and TRNA are the mtDNA-encoded tRNA genes. Arrows correspond to the most commonly occurring mtDNA variants located near poly-C tracts. Y-axis of SNV and indel plots correspond to the number of variant-sample pairs observed at each site across both biobanks. Dotted line corresponds to 500 instances of the observed variant.

We applied mtSwirl^4^, a pipeline for calling mtDNA abundance and heteroplasmic variation (Methods), to whole-genome sequencing (WGS) data from 736,038 diverse individuals in AoU and UKB, representing an approximately 3× increase in sample size compared with ref. ^4^. mtSwirl reconstructs each individual’s mtDNA to serve as a ‘self-reference’ against which low levels of heteroplasmic variants can be more accurately called^4^. After quality control, we called a total of 19,051,526 variants (Methods) from 620,385 individuals. Of the 1,151,297 heteroplasmic variants (Supplementary Note 1), 754,108 were indels and 397,189 were SNVs (Extended Data Fig. 1).

**Extended Data Fig. 2:**
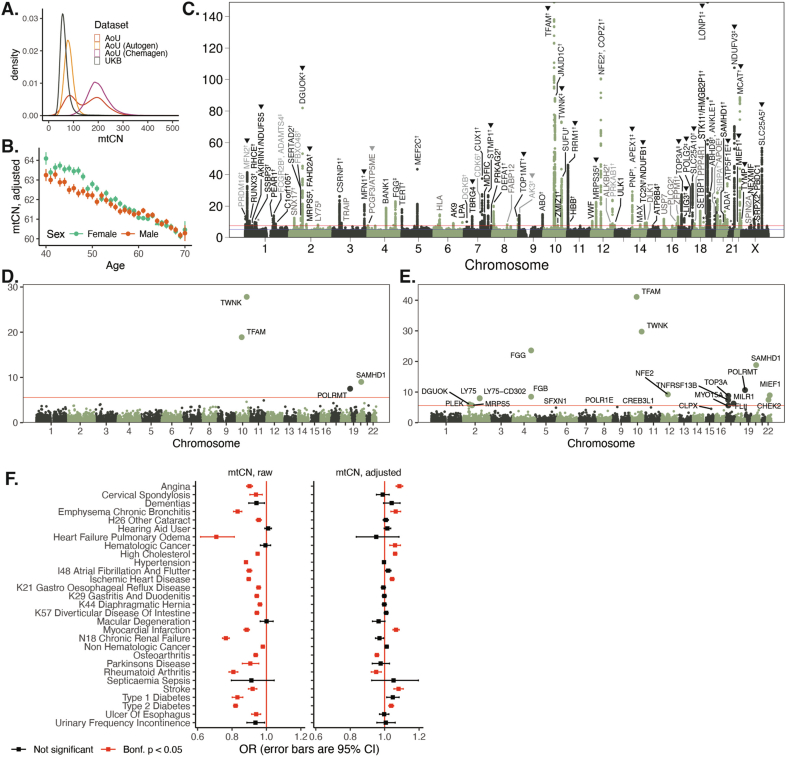
Nuclear genetic and phenotypic correlates of mtDNA copy number across UKB. **A**. Distribution of mtCN_raw_ across AoU (aggregate and stratified by DNA extraction kit) and UKB. **B**. mtCN_adj_ as a function of age and sex in UKB. Each point represents mean mtCN_adj_ per bin. Error bars represent +/− 1SE. *n* = 401,385 individuals. **C**. GWAS of mtCN_adj_ across 398,250 individuals in UKB. Gene assignments were made based on a combination of manual curation, fine-mapping, and nearest gene. Asterisks above chromosomes 1, 18, 19, 20, 21 represent DOCK7, BCL2, GP6, SNX5, RUNX1 respectively. Grey annotations correspond to loci that decreased in significance after correction for technical variables and blood composition. Arrows point to genes thought to produce protein products that localize to mitochondria. † = gene nearby fine-mapped variant with PIP > 0.1 and ‡ = gene with fine-mapped variant at PIP > 0.1 causing a protein sequence change. **D**. Gene-based testing for association between ultra-rare (AF ≤1 * 10^−4^) missense and LoF variation and mtCN_adj_. **E**. Gene-based testing for association with mtCN_adj_ using the Cauchy combination method. **F**. Impact of adjustment of mtCN for technical and blood composition covariates on correlations with 29 common curated case/control disease traits. Each point represents an odds ratio (OR) for association. OR is computed using logistic regression including covariates for sex, age, ancestry, and haplogroup. Error bars represent 95% CI. Per-test sample sizes and test statistics can be found in Supplementary Table 10. P-values are two sided and adjusted using the Bonferroni method.

**Extended Data Fig. 3:**
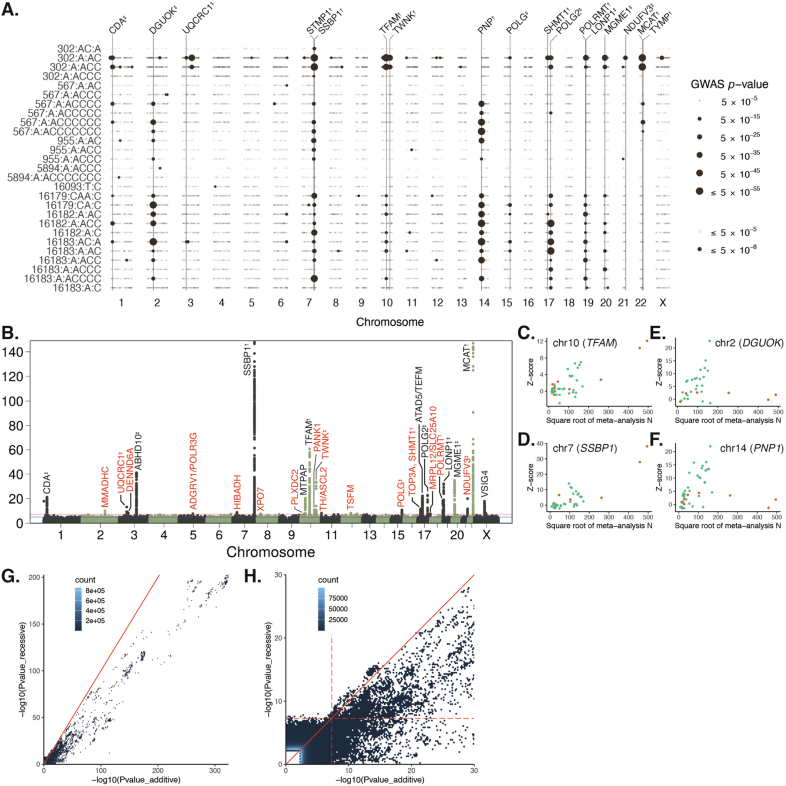
Heterogeneity in nuclear genetic influences on common heteroplasmic mtDNA sites. **A**. Case-only GWAS for common heteroplasmies for all traits that show >=1 genome-wide significant association. **B**. GWAS for chrM:302:A,AC heteroplasmy (*n* = 244,879). Genes were identified as nearest or via manual curation. Red genes correspond to those not identified with the previous analysis in ~68,500 individuals. In both **A** and **B**, † = gene nearby fine-mapped variant with PIP > 0.1 and ‡ = gene with fine-mapped variant at PIP > 0.1 causing a protein sequence change. Comparison of square root sample size with association Z-scores for **C**. TFAM, **D**. SSBP1, **E**. DGUOK, and **F**. PNP locus. In **C-F**, orange corresponds to chrM:302 traits and green are other common heteroplasmies (567, 955, 5894, 16179–16183). **G**. 2D histogram to compare between recessive and additive p-values from the cross-ancestry and cross-biobank meta-analysis for heteroplasmy traits. **H**. Zoomed in view of panel **G**. Dotted lines are 5e-8 p-value threshold. Diagonal line is y = x.

To benchmark our callset, we performed GWAS of mtDNA copy number (mtCN) and individual common heteroplasmic variants and compared them with the results of our previous study^4^. GWAS for blood-composition-adjusted mtCN in UKB (mtCN_adj_; Supplementary Note 2) across 398,250 individuals identified 107 loci (Extended Data Fig. 2c and Methods), many of which were corroborated by fine-mapping and gene-based rare-variant testing (Extended Data Fig. 2d,e). We replicated 44 of 46 loci from our previous mtCN_adj_ GWAS^4^ (*n* = 163,372) at genome-wide significance (GWS) and discovered 63 further associations (for instance near *LIG3* and *TBRG4*). As in previous work^4,11^, adjustment for blood cell composition eliminated or reversed the direction of most associations between reduced mtCN and increased rates of age-related diseases (Extended Data Fig. 2f). GWAS for heteroplasmy of each of 78 common mtDNA variants, most of which were indels (Extended Data Fig. 1), identified 163 associations across 60 nuclear loci (Extended Data Fig. 3a). We replicated virtually all previously identified associations^4^ and identified many more hits. For example, we report associations between chrM:302:A:AC (the most common heteroplasmy in humans) and nucDNA variants near *TSFM*, *TOP3A*, *PANK1* and *UQCRC1*; *UQCRC1* is supported by a fine-mapped missense variant (Extended Data Fig. 3b). Across mtDNA sites, *DGUOK* and *PNP* were associated with most heteroplasmies (*N* < 42,000) except for chrM:302 (*n* = 244,879) (Extended Data Fig. 3a–f), implying differences in nuclear control of heteroplasmy across mtDNA sites beyond the effects of power (Supplementary Note 3). Recessive GWAS revealed no new loci (Extended Data Fig. 3g,h).

## Heteroplasmic mtSNVs reflect replication error

**Fig. 1:**
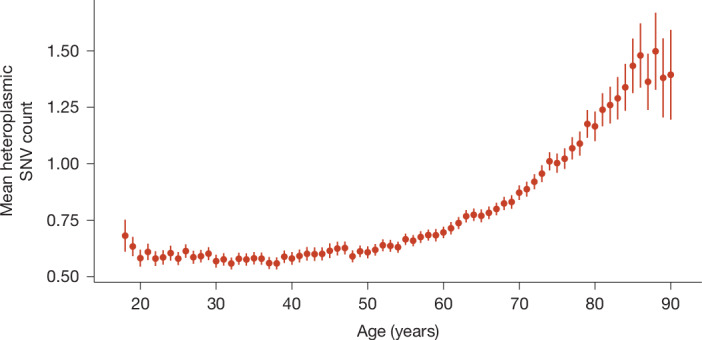
Age accumulation of mtSNVs in AoU (*n* = 236,749 individuals). Points represent means, error bars represent 95% confidence intervals. See Extended Data Fig. 4a for the corresponding analysis in UKB.

**Extended Data Fig. 4:**
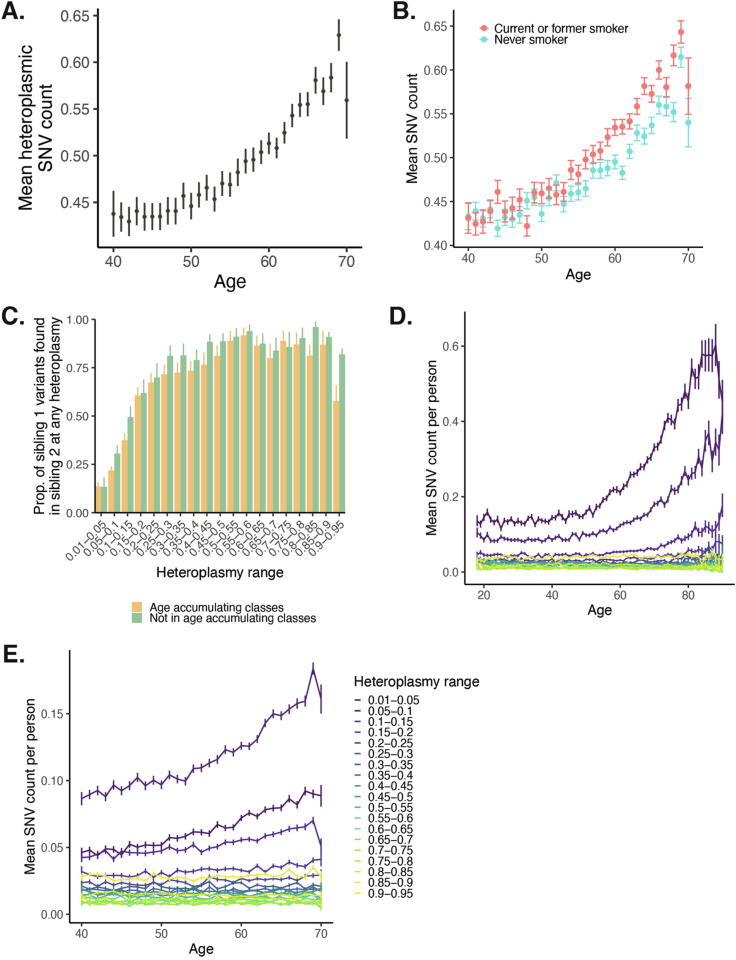
Correlates of age accumulation of mtSNVs. **A**. Age accumulation of mtSNVs in UKB (*n* = 343,266 individuals). Error bars represent 95% CI. See Fig. 1 for a similar analysis in AoU. **B**. Relationship between age and mtDNA heteroplasmy count as a function of smoking status (*n* = 373,823 individuals). **C**. Degree of variant sharing between siblings as a function of heteroplasmy in UKB. Each bar represents the proportion of one sibling’s variants found in the other sibling at any level. Color represents SNVs that are in age accumulating classes (yellow) versus those that are not. Results computed using *n* = 2,821 variants. Mean heteroplasmic SNV count as a function of age broken down by variant heteroplasmy range in **D**. AoU (*n* = 236,749 individuals) and **E**. UKB (*n* = 343,266 individuals). For panels **D** and **E**, color corresponds to heteroplasmy range legend next to panel **E**. For panels **B-E**, error bars are +/− 1SE.

Focusing on heteroplasmic mtSNVs in AoU, we observed mutation accrual particularly after the age of 60 years (Fig. 1). We found a similar mtSNV accumulation in UKB (Extended Data Fig. 4a). This accumulation occurred regardless of smoking status; however, heteroplasmic SNVs were more abundant in smokers, as described previously^12^ (Extended Data Fig. 4b).

**Fig. 2:**
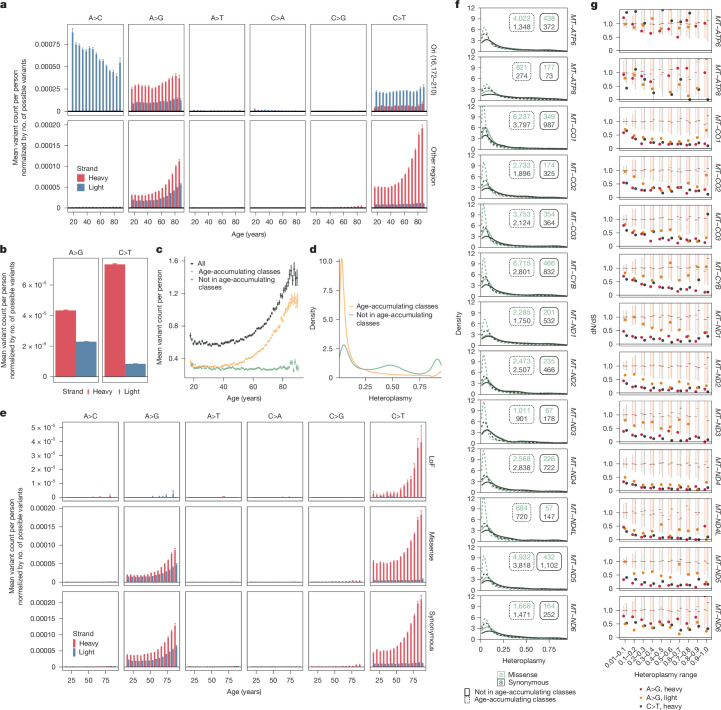
Age-accumulating mtSNVs show a stark strand bias, tend to occur at lower heteroplasmy and are more neutral than other variants. **a**, Normalized mean mtSNV count as a function of variant location, strand, class and age. **b**, Normalized mean mtSNV count as a function of strand and variant class. **c**, Mean mtSNV count as a function of age for all variants, age-accumulating class variants (C>T heavy and A>G heavy and light) and other variants. **d**, Heteroplasmy distribution across variants and individuals for age-accumulating classes only and all variants. **e**, Normalized mean mtSNV count for coding variants as a function of strand, class, age and consequence. **f**, Heteroplasmy distributions for missense (green) versus synonymous (black) variants within mtDNA protein coding genes, stratified by age-accumulating class (dashed versus solid). Insets are corresponding sample sizes. **g**, dN/dS estimates (dots) as a function of heteroplasmy, gene and mutation class for age-accumulating class variants in mtDNA protein coding genes. Dashes indicate the median dN/dS of null draws; error bars are 95% confidence intervals under null, computed non-parametrically as the 2.5–97.5% range of null draws. In **a**–**c** and **e**, error bars indicate ±1 s.e. For **a**, **c** and **e**, total *n* = 236,749 individuals. For **b** and** g**, total *n* = 237,500 individuals. All panels in this figure use AoU data; see Extended Data Fig. 5 for UKB.

**Extended Data Fig. 5:**
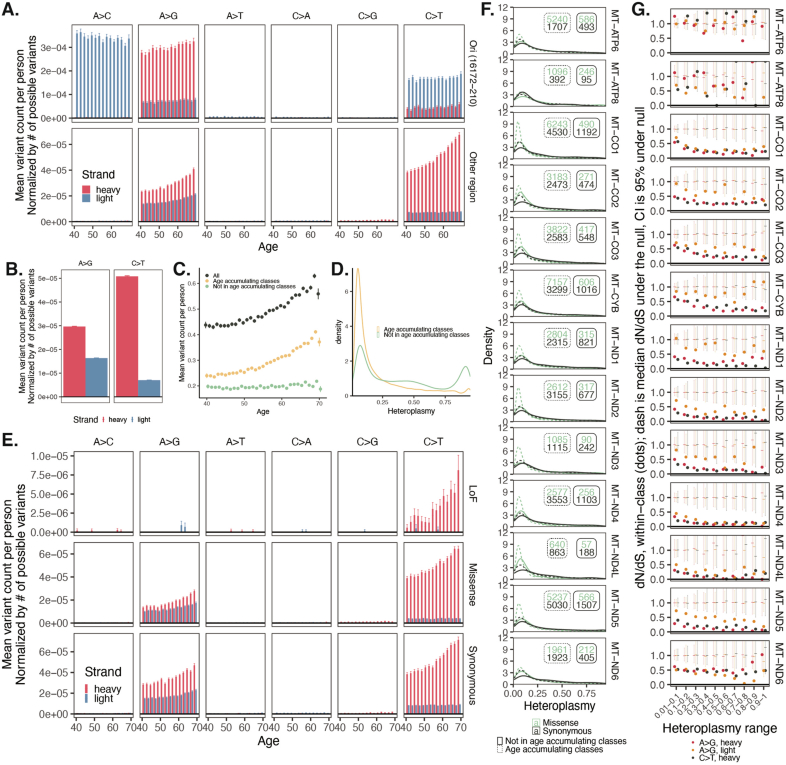
Mutational spectrum and markers of selection for age-accumulating mtSNVs in UKB. **A**. Normalized mean mtSNV count as a function of variant location, strand, class, and age. **B**. Normalized mean mtSNV count as a function of strand and variant class. **C**. Mean mtSNV count as a function of age for all variants, age-accumulating class variants (C > T heavy and A > G heavy and light), and other variants. **D**. Heteroplasmy distribution across variants and individuals for age-accumulating classes only and all variants. **E**. Normalized mean mtSNV count for coding variants as a function of strand, class, age, and consequence. **F**. Heteroplasmy distributions for missense (green) versus synonymous (black) variants within mtDNA protein coding genes, stratified by age accumulating class (dotted versus solid). Insets are corresponding sample sizes. **G**. dN/dS estimates (dots) as a function of heteroplasmy for age-accumulating class variants in mtDNA protein coding genes. Dashes are median of null draws; error bars are 2.5%–97.5% range of null. For panels **A**, **B**, **C**, **E**, error bars are +/− 1SE. For panels **A, C, E**, total *n* = 343,266 individuals. For panels **B, G**, total *n* = 382,505 individuals. All panels of this figure use UKB data; see Fig. 2 for the corresponding analyses in AoU.

We first visualized mtDNA mutations by variant type and strand. mtDNA contains a ‘heavy strand’ with more purines and a ‘light strand’ with more pyrimidines. Replication begins on the light strand at the origin of replication (Ori). We excluded Ori, as it has been shown to have a different mutational spectrum from the coding mtDNA^9^. Outside Ori, most variants were transitions (Fig. 2a,b and Extended Data Fig. 5a,b) with a striking strand bias: C>T and A>G variants occurred far more frequently on the heavy strand than on the light strand.

Classically, mutations in mtDNA have been thought to arise owing to oxidative stress^7^; however, growing evidence over the past decade^8,9,13^ has suggested that replication-linked errors may better explain heavy-strand-biased transition mutations. Under the strand displacement model, once replication of the light strand begins at Ori, the non-template heavy strand persists in a single-stranded state until replication progresses through approximately two-thirds of the molecule^14^. A bias towards heavy strand C>T and A>G mtSNVs has been observed in human brain and tumour samples and attributed to deamination from the protracted time that the heavy strand spends in a single-stranded state^9,15^. By contrast, oxidative damage predominantly produces C>A transversions due to guanosine oxidation^16–18^. Consistent with findings in human brain and tumours^8,9^, our observation of heavy-strand-biased C>T and A>G mutations with little C>A in blood suggests replication-related errors with little oxidative damage.

**Extended Data Fig. 6:**
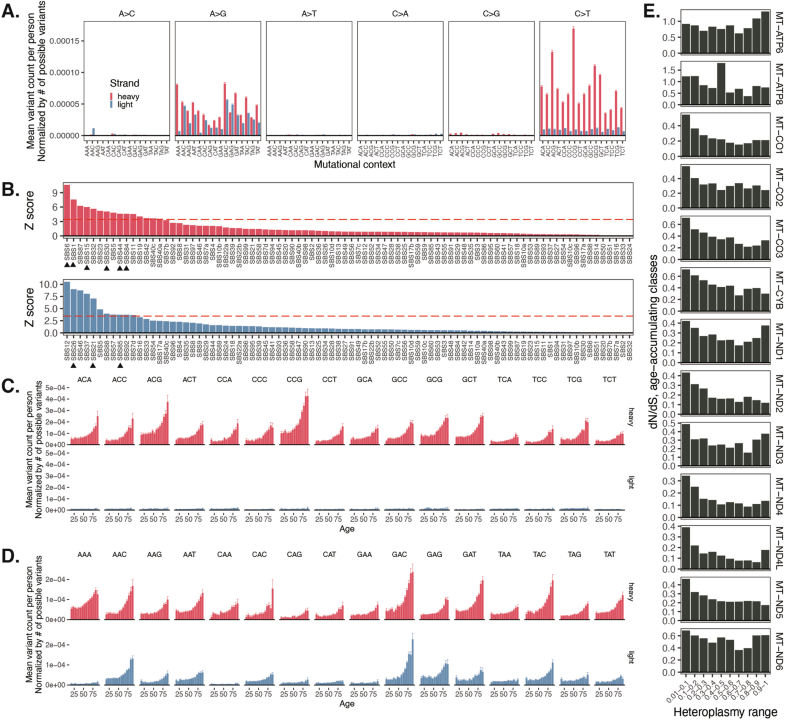
Mutational signature and selection analysis of mtSNVs in AoU. **A**. Normalized mean mtSNV count as a function of variant class, strand, and tri-nucleotide context. Context is determined by the 5’ and 3’ base in a strand-specific way. *n* = 237,500 individuals. **B**. Z-scores from p-values obtained via linear regression of mtDNA mutation spectrum onto all cancer nuclear single base substitution signatures. Upper plot represents heavy strand, lower represents light strand. Line corresponds to Bonferroni-corrected significance threshold at p < 0.05. Triangles represent significantly-associated SBS spectra thought to be related to impaired DNA damage repair and deamination. Age-accumulation of normalized mean mtSNV burden as a function of strand and trinucleotide context for **C**. C > T and **D**. A > G variants. For panels **A**, **C**, and **D**, error bars correspond to +/− 1 SE. For panels **C** and **D**, *n* = 236,749 individuals. **E**. dN/dS point estimates stratified by gene and heteroplasmy bin. Only age-accumulating class variants were used as input to dN/dS estimation, and estimation was performed separately for each heteroplasmy range and biobank. A 192-rate parameter substitution model was used as part of the *dNdScv* package. All panels of this figure use AoU data; see Extended Data Fig. 7 for the corresponding analyses in UKB.

**Extended Data Fig. 7:**
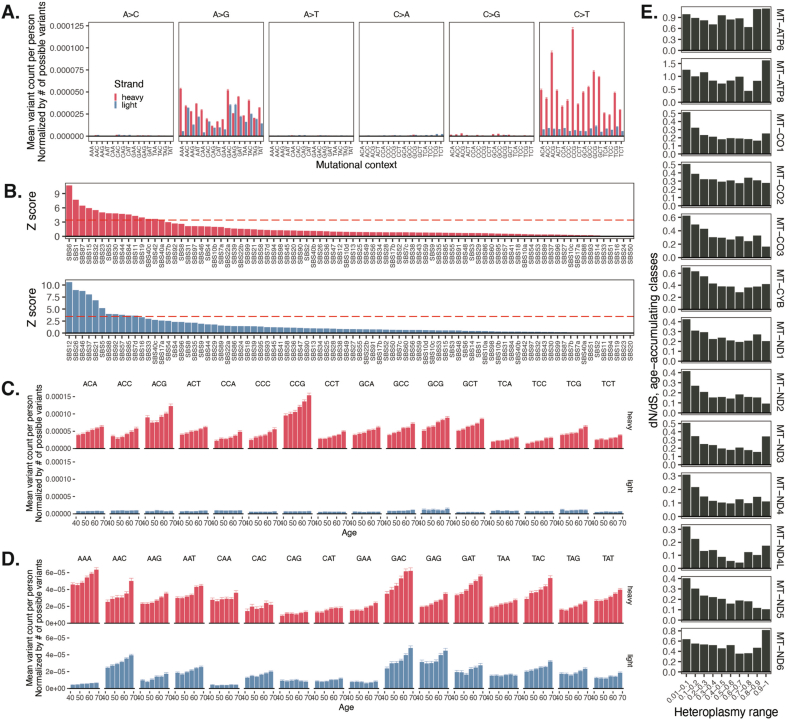
Mutational signature and selection analysis of mtSNVs in UKB. **A**. Normalized mean mtSNV count as a function of variant class, strand, and tri-nucleotide context. Context is determined by the 5’ and 3’ base in a strand-specific way. *n* = 382,505 individuals. **B**. Z-scores from p-values obtained via linear regression of mtDNA mutation spectrum onto all cancer nuclear single base substitution signatures. Upper plot represents heavy strand, lower represents light strand. Line corresponds to Bonferroni-corrected significance threshold at p < 0.05. Age-accumulation of normalized mean mtSNV burden as a function of strand and trinucleotide context for **C**. C > T and **D**. A > G variants. For panels **A**, **C**, and **D**, error bars correspond to +/− 1 SE. For panels **C** and **D**, *n* = 343,266 individuals. **E**. dN/dS point estimates stratified by gene and heteroplasmy bin. Only age-accumulating class variants were used as input to dN/dS estimation, and estimation was performed separately for each heteroplasmy range and biobank. A 192-rate parameter substitution model was used as part of the *dNdScv* package. All panels of this figure use UKB data; see Extended Data Fig. 6 for the corresponding analyses in AoU.

Although mechanisms of mtDNA and nucDNA replication are distinct, we briefly explored whether our observed mtDNA mutation spectrum had parallels with nucDNA. Our observed pattern of mtSNVs was most correlated with known nuclear single-base substitution signatures attributed to nucDNA damage repair defects (for instance, mismatch repair) (Extended Data Figs. 6a,b and 7a,b). On the other hand, nuclear mutation signatures attributed to oxidative damage^19–21^ were not correlated with our observed mtDNA mutational signature.

Only C>T mutations (on the heavy strand) and A>G mutations (on both strands) accumulated with age (Fig. 2a and Extended Data Fig. 5a), whereas heteroplasmic SNVs outside these classes did not (Fig. 2c and Extended Data Fig. 5c). In contrast to A>G and C>T heavy strand mutations, for which all trinucleotide contexts showed age accumulation (Extended Data Figs. 6c,d and 7c,d), only a subset of A>G light strand contexts showed age accumulation. A contributing mechanism may be polymerase error, which can produce transitions^22^ and could be context-specific and strand independent.

We previously showed that mtSNV heteroplasmy tends to be somatic and not maternally transmitted^4^. Here we wanted to know whether this observation extended to age-accumulating class variants (that is, A>G on both strands and C>T on heavy strand). SNVs with high heteroplasmy (higher than 0.2) showed a greater than 75% chance of being found in both siblings, whereas low-heteroplasmy (0.2 or lower) variants had a less than 30% chance of being shared between siblings (Extended Data Fig. 4c). To control for the possibility that sibling-sharing could have been present but below our detection threshold, we repeated this analysis for chrM:302:A:AC, which tends to be maternally inherited^4^; we found that more than 60% of variants at heteroplasmy less than 0.2 showed sibling-sharing. Most age-accumulating class variants had heteroplasmy less than 0.2, substantially less than that of non-age-accumulating class variants (Fig. 2d and Extended Data Figs. 4d,e and 5d). Overall, these results indicate that age-accumulating class mtSNVs may have lower heteroplasmy and are more likely to be somatic than non-age-accumulating class variants.

## Age-accumulating mtSNVs appear neutral

We next sought to determine whether positive selection shaped the observed pattern of heteroplasmic mtSNV accrual. We observed a similar strand bias among age-accumulating class variants regardless of whether the variant was missense or synonymous (Fig. 2e and Extended Data Fig. 5e), arguing against selection as a central driver. Missense variants had left-shifted heteroplasmy distributions relative to synonymous variants for all genes, suggesting selection against coding variants at higher levels of heteroplasmy (Fig. 2f and Extended Data Fig. 5f). A metric traditionally used to quantify the degree of selection acting on variation is the normalized ratio between non-synonymous and synonymous variation (dN/dS), where a ratio less than 1 suggests negative selection, approximately 1 suggests neutrality and greater than 1 suggests positive selection. In mtDNA, this metric must account for the underlying mutational process (which is strand- and mutation-class-specific) and codon composition. Accordingly, we used two methods to estimate and interpret dN/dS: first, a non-parametric approach recently used in mtDNA^23^; second, a parametric approach accounting for strand and mutation class^24^ (Methods). We found that dN/dS among age-accumulating variants was closest to neutral at low heteroplasmy and fell as heteroplasmy rose in all genes except *MT-ATP6*, *MT-ATP8* and *MT-ND6* (Fig. 2g and Extended Data Figs. 5g, 6e and 7e). These results indicate that purifying selection against deleterious alleles may increase with higher levels of heteroplasmy, consistent with the ‘heteroplasmy threshold effect’ in which high levels of heteroplasmy are required before phenotypic effects of the mutation are observed^25–28^. The variants that accumulate with age, however, tend to be low-heteroplasmy and are probably functionally neutral.

## GWAS of mtSNV burden implicates CH

**Fig. 3:**
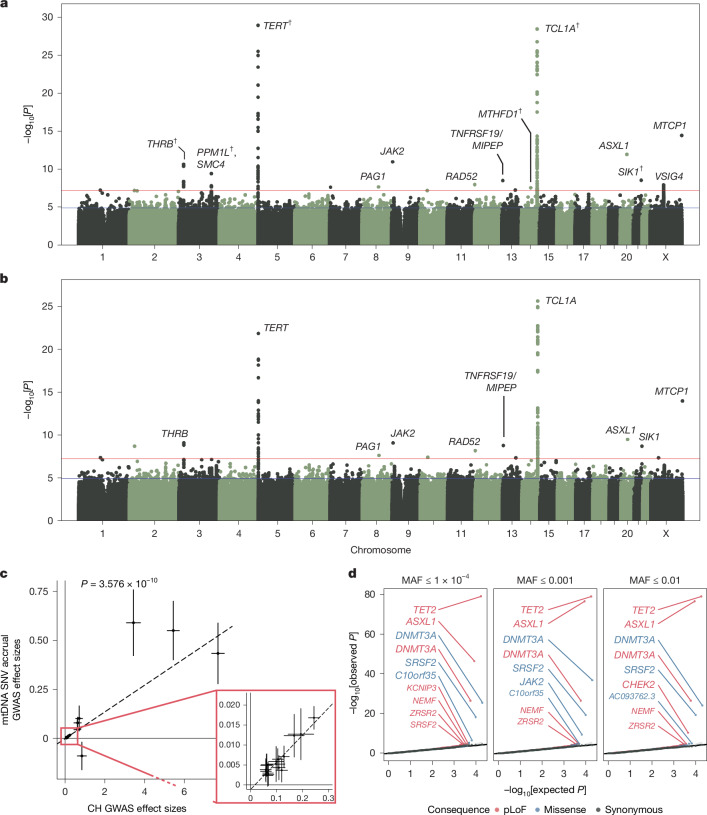
Nuclear genetic analysis of mtSNV burden identify drivers and correlates of CH. **a**, Common variant GWAS meta-analysis between AoU and UKB for age-accumulating heteroplasmic mtSNV burden (maximum *n* = 547,947 individuals). Labelled genes identified as nearest or through curation. **b**, GWAS as in **a** but excluding any sample with detected CH (maximum *n* = 530,443 individuals). In **a** and **b**, red lines correspond to the 5 × 10^−8^ GWS threshold. **c**, Comparison of GWAS effect size estimates for mtSNV count trait versus those for CH (maximum *n* = 363,475 individuals) as part of bidirectional Mendelian randomization. Inset represents zoom near origin. Dotted line indicates the inverse-variance weighted linear regression line, with *P* values from two-sided test for a non-zero beta. Points represent effect sizes. CH effect sizes are log-odds ratios, and mtSNV accrual effect sizes are linear regression betas. Alleles were set such that lead variants for CH were risk-increasing. Error bars are 95% confidence intervals. **d**, Quantile–quantile plots for gene-based SKAT-O tests of association between variants of various classes (colours) and MAF cutoffs (panels) and SNV count. Genes labelled with large text passed Bonferroni GWS; smaller-text labels correspond to genes passing a 10% false discovery rate cutoff using the Benjamini–Hochberg procedure. ^†^Gene nearby fine-mapped variant with PIP > 0.1.

**Extended Data Fig. 8:**
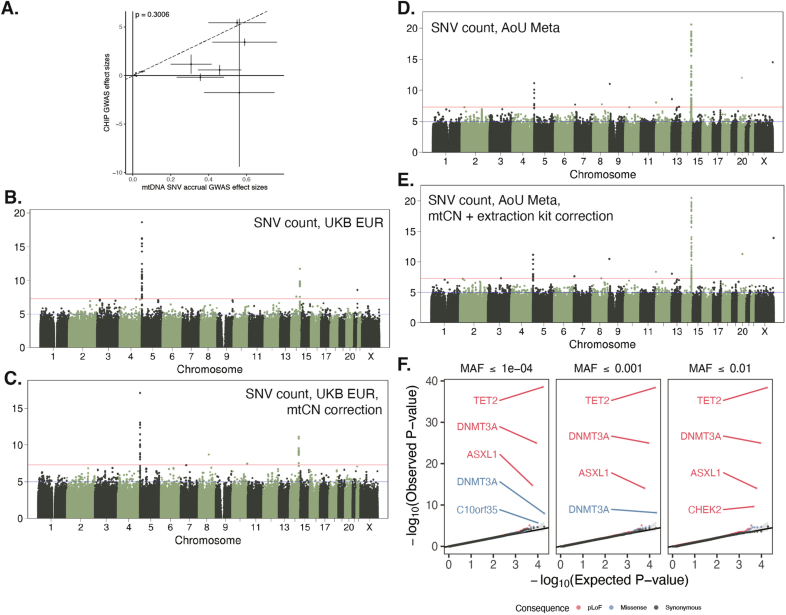
Evaluation of the common and rare variant landscapes of mtSNV accrual. **A**. Detection of reverse causation between CH (max *n* = 363,475 individuals) and mtSNV accrual (max *n* = 547,947 individuals) using MR. Dotted line is inverse variance weighted linear regression line with p-value representing a two-sided test for a non-zero beta. Points represent effect sizes. CH effect sizes are log-odds ratios and mtSNV accrual effect sizes are linear regression betas. Alleles are set such that lead variants for mtSNV accrual are risk-increasing. Error bars are 95% CI. Common variant GWAS of age accumulating heteroplasmic SNV burden in UKB EUR with **B**. the usual covariates (sex, age, PCs, haplogroups) and **C**. with an additional covariate for mtCN_adj_. Common variant GWAS with cross-ancestry meta-analysis of age accumulating heteroplasmic SNV burden in AoU with **D**. the usual covariates (sex, age, PCs, haplogroups, site) and **E**. with additional covariates for mtCN and extraction kit. **F**. Quantile-quantile plots showing SKAT-O p-values from gene-based association testing of heteroplasmic SNV count after individuals with identified CH were removed. Analysis shows a meta-analysis between UKB and AoU cross-ancestry meta-analyses. Various variant classes and AF cutoffs are denoted by colors and panels respectively. See Fig. 3d for corresponding analysis with all samples retained.

Next, we sought to define the mechanism driving the age-dependent accumulation of mtSNVs. We performed GWAS using mtSNV count as the phenotype (*n* = 547,947). Surprisingly, we found genome-wide significant associations near *TERT*, *TCL1A*, *THRB*, *SMC4*, *VSIG4* and others (Fig. 3a), almost none of which corresponded to nuclear-encoded mitochondrial proteins. This was in stark contrast to our GWAS for mtCN and mtDNA heteroplasmic indels, which implicated mtDNA replication machinery. The associated variants tended to fall near genes involved in nucDNA repair (for instance, *TERT*, *RAD52* and *SMC4*) and haematopoietic cell proliferation (such as *TCL1A*, *MTCP1* and *THRB*). Most have been found to be associated with CH, and *TERT* and *SMC4* have been found to have the most significant genetic associations with CH^2^. *TCL1A* has been linked to heterogenous effects on *DNMT3A*-, *TET2*-, and *ASXL1*-driven CH^2^, and the exact *TCL1A* variant we identified has been implicated in the haematopoietic clonal expansion rate^29^. Similar to those identified in our mtSNV GWAS, very few of the genes implicated in CH GWAS encoded mitochondrial proteins^2^. We identified ‘CH carriers’ in UKB and AoU on the basis of the presence of putative somatic variants within known nuclear driver genes (Methods) and found that most of the identified GWS associations for mtSNV count persisted after removal of 17,504 individuals labelled as CH carriers, albeit with slightly reduced significance (Fig. 3b). We next performed Mendelian randomization between mtSNV burden and CH. Across virtually all variants associated with CH at GWS^2^, we observed a positive correlation with corresponding effect sizes for mtSNV burden (Fig. 3c and Methods), indicating that CH increases mtSNV burden. The converse was not true (Extended Data Fig. 8a), suggesting that mtSNV burden does not affect CH risk. As our method for quantification of mtSNVs relies on a cutoff dependent on mtDNA coverage (Methods and Supplementary Note 1), as a sensitivity analysis we repeated our GWAS in UKB and AoU including mtCN as a covariate. We found minimal changes to the observed genetic architecture (Extended Data Fig. 8b–e).

## CH driver mutations predict mtSNV burden

To complement our common variant analysis, we performed a rare-variant association study (RVAS) to identify rare variants in the nuclear genome (Methods) that co-occurred in samples with a high burden of heteroplasmic mtSNVs. We found that predicted loss-of-function and missense variants in several genes, including *ASXL1*, *DNMT3A*, *TET2*, *SRSF2*, *JAK2*, *CHEK2* and *NEMF* (Fig. 3d), tended to correlate with high mtSNV burden. Most of the genes identified through RVAS were classical CH driver genes in which somatic variants are used to identify individuals with CH^2^. Recent work has identified evidence of positive selection in *CHEK2* in CH, suggesting that it can act as a non-classical driver as well^30^. Our results indicated that CH may contribute to changes in detected mtSNV burden, in turn suggesting that our gene-based tests were, in many cases, identifying somatic driver mutations in nucDNA associated with mtSNV burden. *ASXL1* and *JAK2* also showed singleton single-variant associations with mtSNV burden (Fig. 3a); the associated nuclear variants were identified only by WGS, raising the possibility that these associations were driven by somatic nucDNA mutations. Although many identified genes are known drivers, *NEMF* is involved in translation but has yet to be linked to CH; thus, it is a potential novel driver gene candidate. Again, removal of individuals labelled as having CH caused a decline in the gene-based test association significance; however, RVAS associations with *DNMT3A*, *TET2*, *ASXL1*, *CHEK2* and *C10orf35* persisted (Extended Data Fig. 8f).

## *TERT* variants link mtDNA, telomeres and CH

Given the rich literature linking the *TERT* gene product telomerase with telomere length and ageing, we next evaluated the evidence for colocalization of the GWAS signal in the *TERT* locus for mtCN (Extended Data Fig. 2c) and mtSNV burden (Fig. 3a). The rs7705526 variant (chr5: 1285859:C:A) had a posterior inclusion probability (PIP) (from fine-mapping) of approximately 1 for mtSNV burden and 0.39 for mtCN, suggesting colocalization (colocalization posterior probability of approximately 0.39). This colocalization was also reported in recent GWAS of CH^2^ (colocalization posterior probability = 1) and telomere length^31^ (colocalization posterior probability = 1). Thus, on the basis of our current work and previous studies, this inherited *TERT* variant: (1) increases mtCN_adj_, (2) increases mtSNV burden, (3) increases CH risk and (4) increases telomere length.

**Extended Data Fig. 9:**
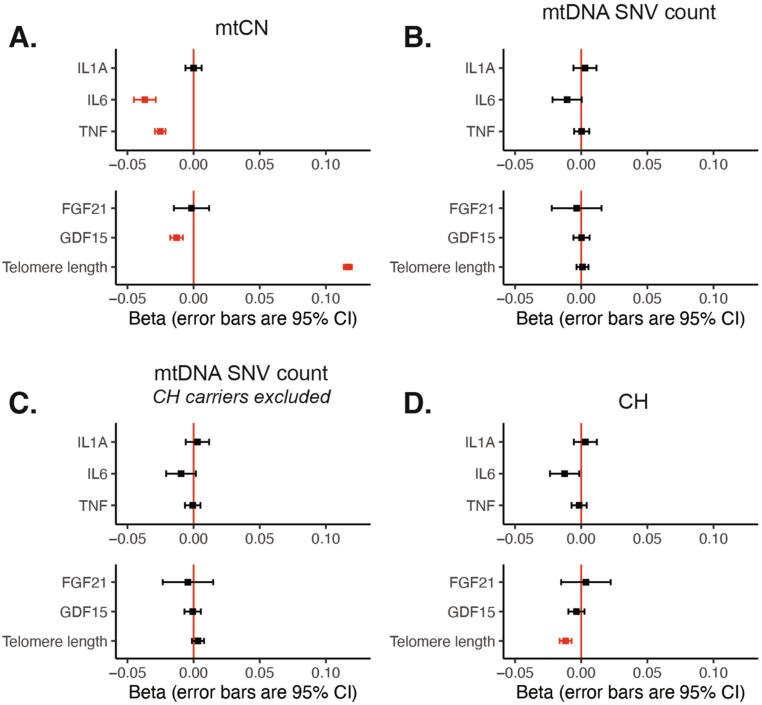
Correlations between mtDNA traits and CH and biomarkers of aging and inflammation. Regression betas of inflammatory biomarkers (upper panel: IL1A, IL6, TNF as measured by proteomics) and aging / reductive stress biomarkers (FGF21 and GDF15 via proteomics and telomere length via PCR) onto **A**. mtCN_adj_, **B**. mtSNV count, **C**. mtSNV count with CH carriers excluded, and **D**. CH. In all analyses, corrections were performed by including age, age^2^, sex, age*sex, age^2^*sex, genetic ancestry, and haplogroup in the association model. Each square point represents a linear regression effect size. Red line is beta = 0. Red points indicate associations that pass Bonferroni multiple testing correction (*n* = 6 tests per analysis). Error bars correspond to 95% CI.

We next explored the phenotypic correlation between key telomere and mtDNA traits in UKB (Methods). After correction for covariates including age, we observed a strong positive correlation between mtCN_adj_ and telomere length (Extended Data Fig. 9a). By contrast, mtSNV burden was not correlated with telomere length (Extended Data Fig. 9b,c). This was not entirely unexpected, as recent work has shown that genetic predisposition to longer telomere length is associated with higher CH, whereas CH subsequently results in a decline in telomere length^32^. Consistent with this, our fine-mapping indicated that the probable causal variant at *TERT* increased telomere length and CH risk. This same variant increased mtSNV count. We speculate that a genetic predisposition for longer telomeres may increase mtSNV count by increasing CH predisposition; however, this effect is masked by the fact that CH itself results in an increase in cell replications, which reduces telomere length but increases heteroplasmic mtSNV count.

We also evaluated the correlation of mtDNA phenotypes with circulating levels of age-associated markers (GDF15, FGF21) and inflammatory cytokines (IL-1A, IL-6, TNF). Even after correction for age, we observed a significant inverse relationship between GDF15 and mtCN_adj_ (Extended Data Fig. 9a), which may indicate a shared underlying mechanism of variation; notably, GDF15 was identified as a correlate of an inborn mitochondrial disease^33^. We also observed that higher mtCN_adj_ was correlated with lower IL-6 and TNF, probably owing to residual effects of inflammatory pathways in blood mtCN_adj_ despite compositional correction. These protein biomarkers did not show significant correlation with mtSNV burden, which probably indicates an independent process (that is, CH due to nucDNA variation rather than expansion due to inflammation).

## mtSNV burden is correlated with blood cancer

**Fig. 4:**
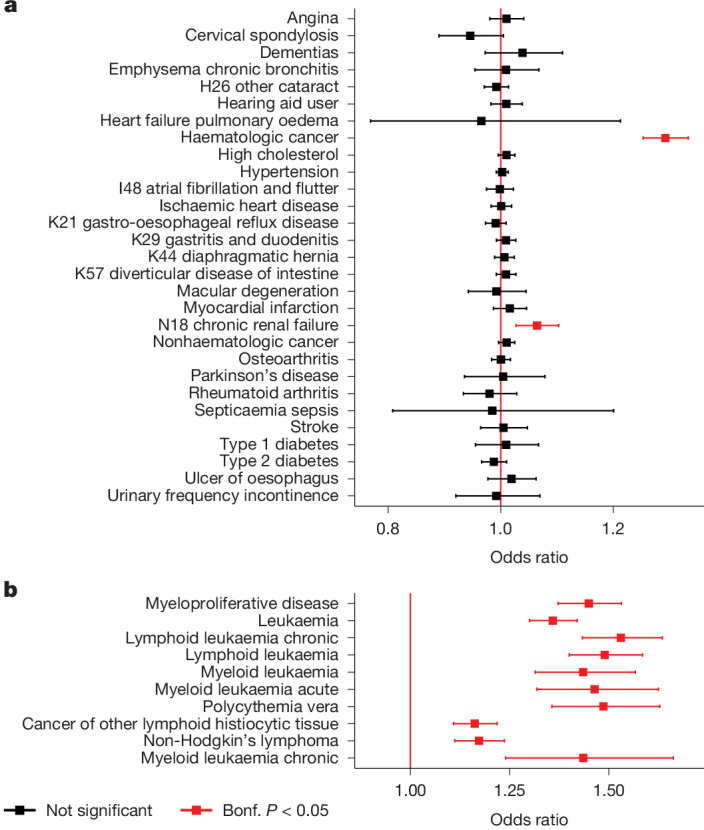
Age-accumulating SNV burden predicts increased risk of haematologic cancer and chronic renal failure when smokers are removed. **a**, Survey of associations with major common diseases. **b**, Significant associations between SNV burden and cancers. Results for all cancers are shown in Extended Data Fig. 10. Points represent odds ratios across both panels. Red indicates an association with Bonferroni-corrected *P* < 0.05. Odds ratios were computed using logistic regression including covariates for sex, age, ancestry and haplogroup. Error bars indicate 95% confidence intervals. Per-test sample sizes and test statistics can be found in Supplementary Tables 8 and 9 for panels **a** and **b**, respectively. Bonf., Bonferroni-corrected; LoF, loss of function.

**Extended Data Fig. 10:**
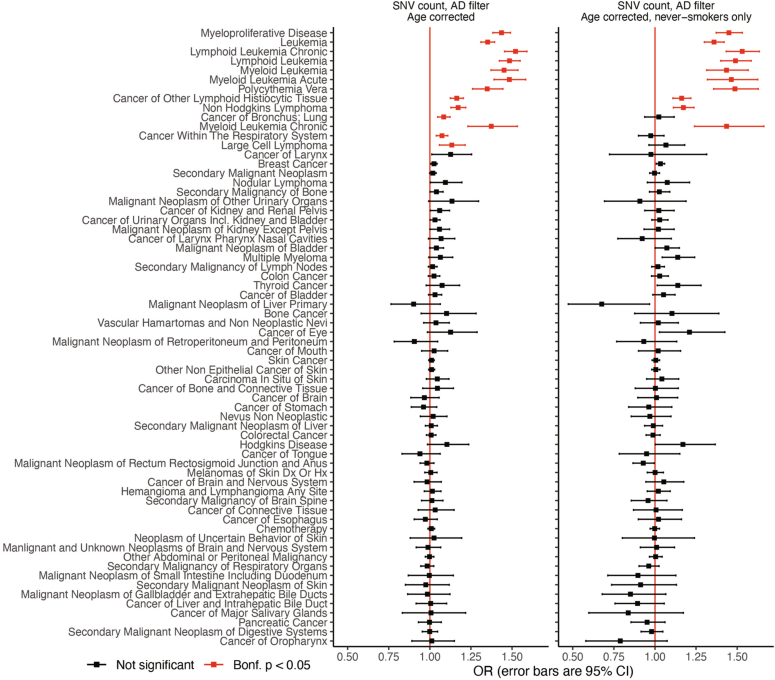
Among all identified cancers, age-accumulating heteroplasmic mtSNV burden is associated with increased risk for a variety of hematologic cancers. Odds ratio is computed using logistic regression including covariates for sex, age, ancestry, and haplogroup. Points represent odds ratios (OR) across both panels. Error bars are 95% CI. Right panel excludes anyone with any recorded smoking history. Red indicates Bonferroni-corrected p-value < 0.05. Per-test sample sizes and test statistics can be found in Supplementary Table 9.

Whether mtSNV burden is associated with disease independent of age is an important question. We focused on 28 common diseases spanning different organ systems. After filtering to never-smokers and applying corrections for age, sex and genetic ancestry group (Methods), we found a strong association for heteroplasmic mtSNV burden only with haematologic cancer; we also found a weak but significant association with chronic kidney disease (Fig. 4a). Notably, we did not observe any associations with other common diseases (Supplementary Table 8). We then extracted cancer-related phenotypes (Methods) and tested for associations with heteroplasmic mtSNV burden. The most substantial effect size was observed for myelodysplastic syndrome, followed by several leukaemia phenotypes (Fig. 4b, Extended Data Fig. 10 and Supplementary Table 9). Ample evidence has linked CH directly to blood cancer risk^34,35^ and even kidney disease^36^. Taken together, our genetic and phenotypic results link the observed age accumulation in mtSNVs in blood with CH.

## mtSNV burden is a sensitive marker of CH

Two lines of evidence demonstrate that mtSNV burden may represent an early and sensitive biomarker of CH. First, GWAS and RVAS for mtSNV burden indicated nuclear germline variants that predisposed individuals to CH, even after removal of the 17,504 individuals with documented CH (Fig. 3a,b). Second, when we removed these individuals from the UKB data (*n* = 11,465), the association between mtSNV burden and haematologic cancer persisted, with an odds ratio of 1.27 (*P* = 3.06 × 10^−47^) versus 1.29 (*P* = 2.33 × 10^−59^) for all samples.

**Fig. 5:**
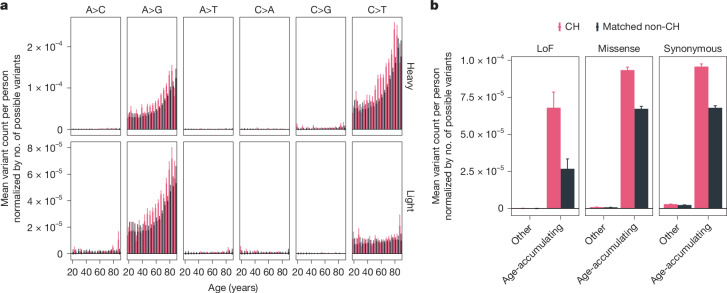
Individuals with identified CH tend to have increased accumulation of age-accumulating mtSNVs relative to the general population and do not show evidence of positive selection. **a**, Normalized mean mtSNV count per person as a function of CH status, strand, variant class and age. **b**, Normalized mean mtSNV count per person as a function of variant consequence and age-accumulating status for CH carriers versus controls. In both panels, error bars correspond to ±1 s.e.. AoU was used for both panels; see Extended Data Fig. 12 for the corresponding analyses in the UKB. Only variants outside the Ori region are shown. For both panels, *n* = 8,266 individuals with CH; these individuals were compared with control individuals, who were selected by identifying 500 random samples of 8,266 age- and sex-matched individuals without CH (Methods).

**Extended Data Fig. 11:**
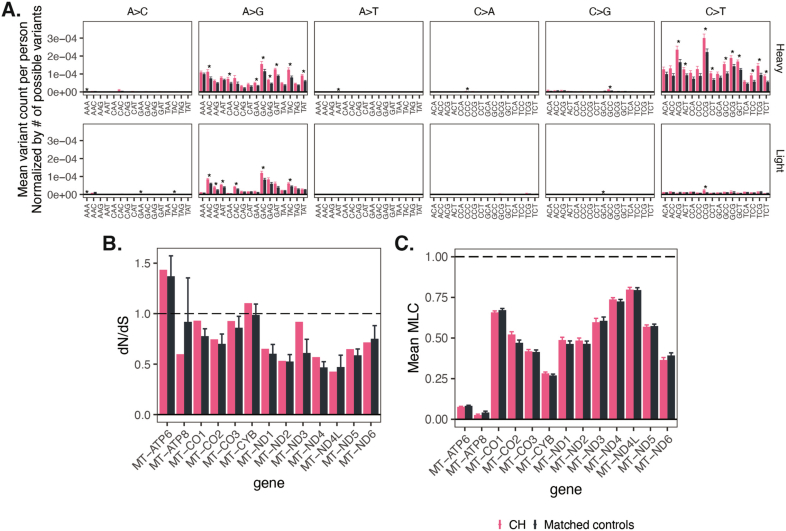
Individuals with CH have more mtSNVs in specific tri-nucleotide contexts relative to the general population with no detected difference in functional consequence. **A**. Normalized mean mtSNV count per person as a function of CH status, variant class, and tri-nucleotide context. * = p < 0.05 for the two-sided test of difference between CH and controls, corrected via Bonferroni method (192 comparisons). P-values can be found in Supplementary Table 11. **B**. dN/dS estimates with non-parametric error estimates for CH carriers versus controls in each coding mtDNA gene among age-accumulating variants. Each bar represents a dN/dS estimate. **C**. Mean MLC metric for age-accumulating variants in UKB for CH carriers versus controls in each coding mtDNA gene. In all panels, error bars correspond to +/− 1SE. All panels of this figure use AoU; see Extended Data Fig. 12 for the corresponding analyses in UKB. Only variants outside the Ori region are shown. For all panels, *n* = 8,266 individuals with CH who are compared to controls, which were selected by identifying 500 random samples of 8,266 age- and sex-matched individuals without CH (Methods).

**Extended Data Fig. 12:**
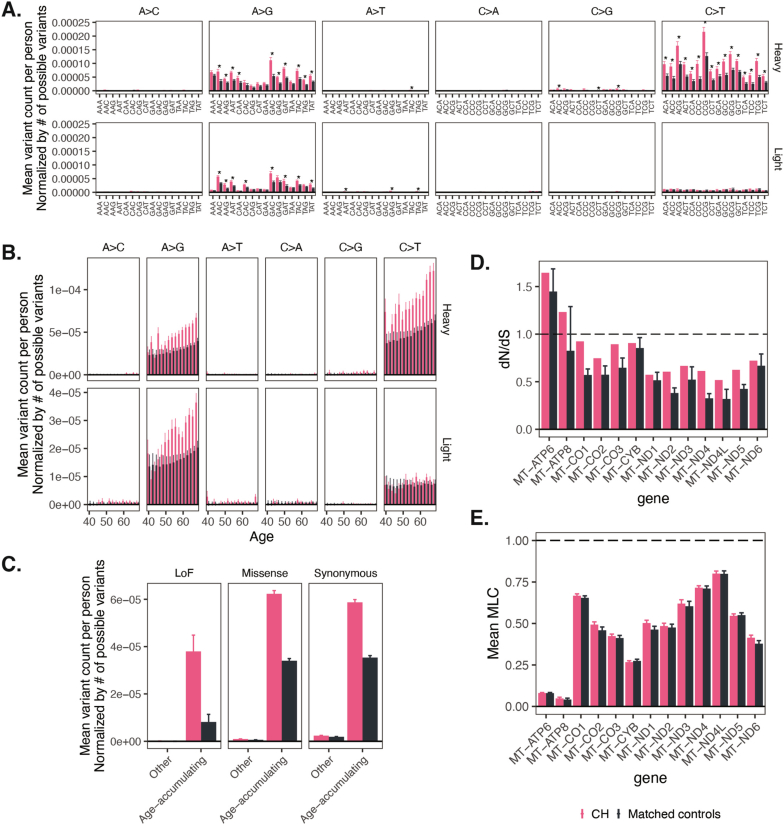
Individuals with identified CH show increased accumulation of mtDNA age accumulating variants relative to the general population in UKB. **A**. Normalized mean mtSNV count per person as a function of CH status, variant class, and tri-nucleotide context. * = p < 0.05 for the two-sided test of difference between CH and controls, corrected via Bonferroni method (192 comparisons). P-values can be found in Supplementary Table 12. **B**. Normalized mean mtDNA variant count per person as a function of CH status, strand, variant class, and age. **C**. Normalized mean mtSNV count per person as a function of variant consequence and age-accumulating status for CH carriers versus controls. **D**. dN/dS estimates for CH carriers versus controls in each coding mtDNA gene among age-accumulating variants. Each bar represents a dN/dS estimate. **E**. Mean MLC metric for age-accumulating variants in UKB for CH carriers versus controls in each coding mtDNA gene. In all panels, error bars correspond to +/− 1SE. All panels of this figure use UKB data; see Fig. 5 and Extended Data Fig. 11 for the corresponding analyses in AoU. Only variants outside the Ori region are included. For all panels, *n* = 10,544 individuals with CH who are compared to controls, which were selected by identifying 500 random samples of 10,544 age- and sex-matched individuals without CH (Methods).

Finally, we directly examined heteroplasmic mtSNV burden among individuals with and without detected CH. Individuals with CH had a similar strand bias for A>G and C>T variants to that of controls, and a concordant mutational spectrum with trinucleotide context (Extended Data Figs. 11a and 12a). This suggested that the underlying mutational mechanism was the same for individuals with and without detected CH. Individuals with CH had significantly more age-accumulating class mtDNA variants and even seemed to have a faster rate of mtSNV accumulation with age (Fig. 5a and Extended Data Fig. 12b) relative to age- and sex-matched individuals without detected CH. As discussed above, only a subset of trinucleotide contexts for A>G light strand mutations showed age accumulation; notably, in A>G light strand contexts, only those that accumulated with age were more abundant in samples with CH versus those without (Extended Data Figs. 6d, 7d, 11a and 12a). Among individuals with CH, we observed a similar increase in the number of age-accumulating class variants for both missense and synonymous variants (Fig. 5b and Extended Data Fig. 12c), arguing against the notion that functional mtDNA variants influence expansion of haematopoietic clones. To evaluate this directly, we used dN/dS and the mtDNA local constraint score, a recently developed positional metric of mtDNA constraint^37^. Neither metric showed strong evidence of positive selection in individuals with CH (Extended Data Figs. 11b,c and 12d,e) relative to controls; dN/dS estimates were substantially greater than 1 for *MT-ATP6*, which is known to have limited constraint^37^. Taken together, these results indicate that mtSNVs may be most likely to be passenger mutations in the setting of an independently arising cellular clonal process in blood.

## Discussion

**Fig. 6:**
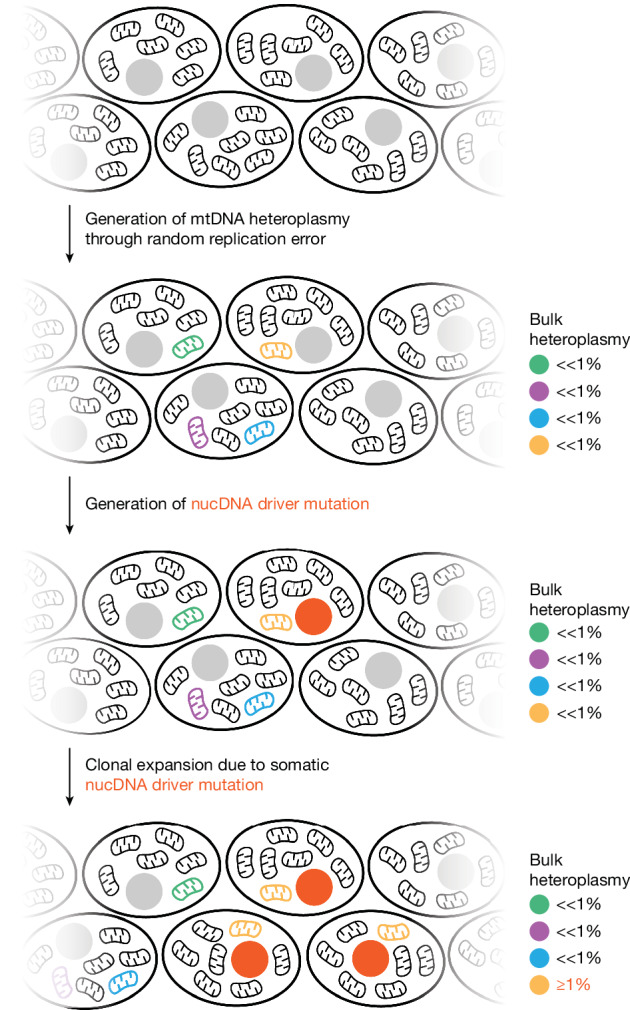
Two-step mechanism by which mtSNV heteroplasmy burden accumulates with ageing. In the first step, cryptic mtDNA variation is generated owing to errors that arise randomly during replication. In the second step, independently generated somatic nucDNA variation drives clonal expansion, carrying cryptic mtDNA variation to detection. Ovals represent cells; large circles represent nuclei.

Age accumulation of human mtSNVs was observed as early as 1999 (ref. ^38^), but both the mutational process underlying this accumulation and why it becomes more apparent later in life have been debated. Here we use large-scale human genetics to elucidate this mechanism in blood (Fig. 6). First, in each cell, mtDNA replication errors generate low levels of ‘cryptic’ variation in the mtDNA. These variants are likely to be functionally ‘silent’ given their low-level heteroplasmy. Then, age-related CH causes expansion of cells carrying somatic mtDNA variants as passengers, rendering them detectable as a function of age. These findings reconcile the mechanistic relationships between observed accrual of age-associated mtDNA mutation and two other prominent signatures of ageing biology, namely CH and *TERT* variants.

We found that heteroplasmic age-accumulating mtSNVs in human blood tended to be transitions with C>T and A>G variants biased towards the heavy strand, consistent with the known mutational signature of mtDNA replication errors. Under the mtDNA strand displacement model, replication of the light strand starts first, leaving the parent heavy strand in a single-stranded state until replication reaches approximately two-thirds of the way through the molecule, and the heavy strand is copied in reverse^14^. This exposed heavy strand is prone to cytosine or adenosine deamination, producing heavy strand C>T and A>G, respectively^9^, in agreement with our data and as seen in *Drosophila*^13^, human tumours^9^ and human brain^8^. The origin of age-accumulating A>G light strand mutations is less obvious. Unlike that of A>G and C>T heavy strand mutations, age accumulation of A>G light strand mutations seems to be trinucleotide context-specific. One possibility is polymerase-γ error, for example, due to proofreading errors or base misincorporation, which tends to produce transition mutations^22,39–41^. By contrast, oxidative stress is thought to predominantly produce C>A transversions through oxidation of deoxyguanosine^16,18^. A>C transversions are also possible if oxidized guanosine is directly incorporated into DNA^17^. In agreement with results obtained in tumour samples^9^ and brain^8^, these transversions were very rare in our data and did not accumulate with age.

Our proposed mechanism (Fig. 6), in which CH acts as an amplifier and allows bulk detection of low-level neutral heteroplasmic mtSNVs with age, is supported by several lines of evidence: (1) individuals with detectable CH tend to have an elevated mtSNV burden; (2) common germline variants in genes such as *TCL1A* and *TERT* that are strongly implicated in CH are also associated with mtSNV accrual; and (3) gene-based analyses identify coding mutations in numerous known CH driver genes as co-occurring in those with high mtSNV burden. These coding mutations probably represent somatic driver mutations for CH that we have now discovered using mtSNV burden as a trait. Previous work in brain required ultradeep sequencing to identify extremely low-heteroplasmy variants that accumulated with age^8^. In whole blood, CH seems to unmask latent variation in mtDNA that is otherwise cryptic and below the threshold of detection. Although the mutation-generating process is likely to be related to mtDNA replication, our analyses suggest that CH heavily shapes the resulting observed pattern of age-associated mtSNV accrual. Our results are compatible with those obtained in models in which somatic mtDNA mutations arise during embryogenesis, early in life^42^ and/or with ageing and are subsequently carried up to detection through CH in older age.

Several lines of evidence indicate that the low-level heteroplasmic mtSNVs in those with detected CH are likely to be passenger mutations rather than drivers. First, CH is identified by detection of known somatic mutations in nuclear driver genes. Given the established mechanisms by which these nuclear driver mutations lead to clonal expansion, it is unnecessary to additionally invoke a separate contribution from these mtDNA variants. Second, we found that both missense and synonymous mtSNVs were similarly increased in CH carriers relative to controls, and we saw no evidence of positive selection for functional mtDNA variation. Third, we found that age-accumulating variants, which are enriched in CH carriers, were strongly enriched for low-heteroplasmy mtSNVs (Fig. 2d). Low-heteroplasmy variation was more likely to be neutral, with a higher dN/dS; by contrast, high heteroplasmy variants were depleted for non-synonymous variation, implying more purifying selection at higher heteroplasmy (Fig. 2f,g). This is consistent with the notion that selective forces on mtDNA variants at the level of the cell increase in strength as a function of increasing heteroplasmy. The heteroplasmy ‘threshold effect’^25,26^ is an established phenomenon in which pathogenic variants do not produce pathologic phenotypes until a sufficiently high heteroplasmy is achieved owing to complementation by wild-type mtDNA molecules. Indeed, in recent work in which dividing cells were engineered to carry a nonsense mutation in *MT-ND4*, fitness effects were dependent on the heteroplasmy level, with low-heteroplasmy cells seeming similar to wild type^27^. Although purifying selection could occur at the level of the organelle even if cell-wide heteroplasmy is low, recent work has indicated a more prominent effect of cell fitness in shaping heteroplasmy^27^. Overall, this suggests that mtSNV burden is unlikely to drive age-related disease (Fig. 4) despite being an excellent ageing biomarker.

Our study focused on age-accumulating passenger mtSNVs, which seem to be neutral and are distinct from pathogenic mtDNA mutations, which cause mitochondrial disease and are under negative selection^4^. Disease-causing mtDNA mutations, which can arise somatically or be maternally transmitted, tend to exhibit high levels of heteroplasmy and disrupt protein expression. Recent single-cell analysis of peripheral blood mononuclear cells from patients with maternally transmitted mtDNA diseases demonstrated purifying selection against high levels of mutant heteroplasmy in some (but not all) cell lineages^43^. An interesting possibility arising from our work is that in some instances of mtDNA disease, high levels of mutant heteroplasmy may be maintained owing to the balancing effects of nuclear genomic driver mutations. Joint sequencing of nuclear and mtDNA genomes in patients with maternally transmitted or sporadic mtDNA mutations could be highly informative.

We propose that mtSNV count in bulk sequencing data could be a sensitive marker of somatic mosaicism that can arise from processes such as CH. Even after removal of all individuals with detected CH, the common germline genetic architecture for mtSNV count was largely unchanged (Fig. 3b), rare variants in CH driver genes still co-occurred with high SNV count, and mtSNV count was still correlated with haematologic cancer. Further, the mtDNA mutational spectrum observed in CH carriers was also seen in those without detected CH (Fig. 5). These findings suggest that there are individuals with undetected CH (for instance, due to unidentified driver mutations) that can be identified on the basis of elevated mtSNV burden, consistent with work showing that CH is universally present at low fraction^44,45^. We suspect the high depth of mtDNA sequencing drives this improved detection of CH, potentially explaining literature suggesting that mtSNV burden is independently associated with blood cancer risk beyond detectable CH^46^. Rare-variant associations with mtSNV burden identified many canonical CH driver genes, non-canonical drivers (such as *CHEK2*) and genes that were otherwise unlinked to CH (for instance, *NEMF* and *KCNIP3*). *NEMF* and *KCNIP3* may represent putative driver genes; *NEMF* is a potential tumour suppressor in lung cancer^47^ and is thought to regulate the DNA damage response^48^ in *Drosophila melanogaster*. Larger sample sizes could enable discovery of more drivers. Notably, a recent method^29^ that used nuclear passenger mutations in CH carriers as a measure of clonal expansion rate also identified the *TCL1A* locus that we report here. This suggests that mtSNV burden, reflecting passenger mtDNA mutations, may integrate information from both the presence or absence of CH and its expansion rate.

Although we have focused on the mechanism underlying the age-related increase in measured mtSNV burden in blood and the role of CH in driving this increase, whether an analogous process (Fig. 6) operates in other tissues is an open question. Mutant mtDNA heteroplasmy has been shown to accumulate with age in other dividing tissues, such as in colon, where functional mtDNA mutations that disrupt electron transport chain activity in colonic crypt stem cells arise and spread throughout the crypt with age^49^. Expansion of mtDNA mutations across crypts through crypt fission into clonal ‘patches’ has also been observed^50^. It is possible that colonic crypt stem cell dynamics resembling CH, in combination with a ‘permissive environment’, contribute to accumulation of missense mtSNVs. In fact, recent work in dividing cells^27^ has shown that certain environments (notably hypoxia) can be permissive for accumulation of high missense or nonsense heteroplasmy. Somatic mtDNA mutations have also been reported to accumulate in non-dividing tissues such as skeletal muscle^51,52^, and it is possible that similar clonal dynamics could occur in stem cell populations^53^.

Our study has some potential limitations. First, there is a risk of contamination of mtSNV count by mtDNA pseudogenes in the nucDNA (NUMTs), which can result in spurious low-heteroplasmy variant calls due to read mismapping. To address this, we constructed heteroplasmy filters to account for the impact of mtCN on NUMT risk, eliminated suspicious variants (Methods) and confirmed that there were no known mtDNA pseudogenes around associated nuclear loci. Long-read sequencing or mtDNA isolation would further reduce contamination. Second, we could not definitively prove whether each variant is inherited or somatic; contaminating germline mutations have been described to bias dN/dS estimates^24^ and would reduce power for detection of nuclear loci that influence somatic mtSNV burden. We used related individuals to show that low-heteroplasmy age-accumulating mtSNVs are predominantly de novo. In addition, dN/dS estimates are unlikely to be differentially biased in individuals with CH versus control individuals. Further, the increase in mtSNV count with CH and age suggests that clone-specific rather than inherited variants increase in heteroplasmy, allowing detection. However, we recognize that, owing to bottleneck effects, some inherited SNVs could exist in heteroplasmy in only some cells, such that they are initially undetectable until clonal expansion^3^. Third, because all analyses occurred in bulk samples, we were unable to evaluate the heteroplasmy distribution of a particular variant across cells in each sample or to determine whether individuals with multiple mtSNVs had a single CH clone carrying all SNVs or multiple clones carrying individual mtSNVs. Single-cell analysis would provide further resolution to elucidate this. Our core results showing the overall mutational spectrum, our observation that higher heteroplasmy variants are subject to higher levels of negative selection, and our nuclear genetic results would not be affected. We anticipate that population-scale, single-cell analysis is likely to be required to further extend our work, identify whether low-heteroplasmy variants in bulk are also low-heteroplasmy in single cells, and uncover interactions between the selective forces on somatic mtDNA variants and CH driver mutations.

## Methods

### Cohorts

#### UK Biobank

The UKB is a prospective cohort of approximately 500,000 individuals from the UK aged 40–70 years at enrolment, described in more detail elsewhere^54^. The resource includes a vast array of information on each individual, including hospital inpatient diagnoses, questionnaires and surveys collected at the time of enrolment, and several aliquots of peripheral blood. These blood samples are used to obtain estimates of blood cell composition, quantitative levels of blood biomarkers and genomic data. The final tranche of WGS data was recently released, covering virtually everyone in the cohort. This is in addition to whole-exome sequencing (WES) and genotyping array data that also exist for each participant. Pertinent to the present study, the final tranche of WGS data was generated using Illumina NovaSeq 6000 sequencing machines across Sanger and the deCODE facility to an average coverage of 32.5×^55^. All WGS and WES data were accessed using the UKB Research Analysis Platform (RAP) under application 31063.

#### All of Us

AoU is a large longitudinal cohort intended to capture the diversity of people living in the USA and includes physical measurements, biospecimen collection and survey data on enrolment, as well as capture of electronic health records^56^. For this study, participant data were accessed from the ‘Genetic determinants of mitochondrial DNA phenotypes (v7)’, ‘Genetic determinants of mitochondrial DNA phenotypes (v7) second 40k’, and ‘Genetic determinants of mitochondrial DNA phenotypes (v7) finish pipeline’ workspaces, all of which use the Controlled Data Repository v.7. This data release incorporated WGS data from 245,394 individuals obtained from peripheral blood and sequenced at Baylor College of Medicine, the Broad Institute and the University of Washington. DNA extraction was performed using one of two kits: Autogen or Chemagen.

### mtSwirl v2: an updated mtDNA coverage estimation and variant-calling approach

To infer mtCN and accurately call variation on mtDNA, we used mtSwirl, a pipeline we have previously described in more detail^4^. In brief, mtSwirl is a scalable pipeline intended for deployment on cloud platforms for analysis of hundreds of thousands of whole-genome sequences. The pipeline involves initial extraction of unmapped reads, as well as a set of 385 nuclear regions in the GRCh38 reference genome with high homology to mtDNA obtained previously through BLAST (putative nuclear regions of mitochondrial homology, NUMT)^4^. These reads are used to perform an initial pass of variant calling using Mutect2 and HaplotypeCaller for mtDNA and nucDNA, respectively, with GATK v.4.2.6.0. High-confidence homoplasmic and homozygous variation, respectively, are then used to construct a per-individual consensus sequence to which all extracted reads are realigned. Alignment is repeated to a shifted version of the consensus sequence to avoid artificial coverage depression along the edges of the mtDNA sequence. mtDNA variant calling is then repeated using Mutect2, and a custom pipeline is used to map all variant calls and per-base coverage estimates back to GRCh38 for output. In parallel, Haplogrep^57^ is used to perform haplogroup inference.

mtSwirl is intended to be usable on any platform that supports the WDL pipeline language. Owing to substantial limitations of parallelization on UKB RAP, we previously released mtSwirlMulti, which runs multiple samples on individual larger machines. As part of this study, to boost efficiency in UKB, we implemented parallelization within each machine for the most computationally intensive tasks in mtSwirl (SubsetBam, ProcessBamAndRevert, HaplotypeCaller, AlignToMtRegShiftedAndMetrics and LiftoverVCFAndGetCoverage). In effect, our updated version of mtSwirlMulti now implements two layers of parallelization, distributing sets of samples across machines and then using multicore machines to efficiently process the sets of samples in parallel. We ran this new version of mtSwirlMulti on approximately 2,000 samples from the previous release and found that the results were completely identical to those obtained previously. mtSwirlSingle is unchanged from before. We also developed new submission pipelines to efficiently automate job submissions in the UKB RAP and AoU. More details are available at our GitHub repository (https://github.com/rahulg603/mtSwirl).

When used on platforms with a Google Cloud backend (such as AoU Researcher Workbench), the pipeline accesses only the portions of the WGS file relevant for the analysis. Despite this, if WGS data are stored in cold storage (for instance, Nearline), data access charges can be substantial.

We deployed mtSwirlMulti on UKB RAP, processing all of the approximately 300,000 new samples in around 3 weeks given the limit of 100 concurrent machines. We ran mtSwirlSingle across approximately 150,000 new samples in AoU over about 1 week. Within each biobank, new results were merged with mtDNA callsets generated in our previous effort across approximately 250,000 individuals^4^, producing a final, per-biobank mtDNA callset of approximately 500,000 individuals in UKB and approximately 250,000 individuals in AoU.

### Sample-level quality control

After variant calls had been completed, samples were removed if:

1. the sample showed evidence of abnormally overlapping homoplasmic mutations;
2. the sample showed evidence of contamination, assessed by testing whether the sample met any of the following criteria: (a) mtDNA contamination estimate > 2% according to HaploCheck 0124 run by mtSwirl^58^, (b) nucDNA contamination estimate > 2% (obtained as a freemix percentage from the genomic metrics table in AoU and quality control (QC) metrics table from UKB) or (c) multiple haplogroup-defining variants identified at relatively low heteroplasmy;
3. the sample was from UKB and was collected during the 2006 pilot analysis.

### Derivation of mtCN

The following formula was used to compute raw mtCN:

$${\rm{mtC}}{{\rm{N}}}_{{\rm{raw}}}=\frac{2\times {\rm{mean}}\,{\rm{mtDNA}}\,{\rm{coverage}}}{{\rm{mean}}\,{\rm{nucDNA}}\,{\rm{coverage}}}.$$

Mean mtDNA coverage was computed and emitted from the mtSwirl pipeline and was generated from alignment of reads to the per-individual consensus sequence using picard CollectWgsMetrics. In AoU, we obtained mean nucDNA coverage from the genomic metrics file provided in the Controlled Tier v.7. For UKB, as this information was not provided, we computed mean nucDNA coverage using the following formula as described previously^4^:

$${\rm{mean}}\,{\rm{nucDNA}}\,{\rm{coverage}}=\frac{{\rm{total}}\,{\rm{mapped}}\,{\rm{reads}}-{\rm{singletons}}-{\rm{mate}}\,{\rm{different}}\,{\rm{chr}}-{\rm{duplicates}}}{{\rm{nucDNA}}\,{\rm{genome}}\,{\rm{length}}}\times {\rm{read}}\,{\rm{length}}.$$

### mtDNA variant QC

For mtDNA variant calls but not mtCN, before variant QC, we also removed any samples with mtCN < 50, as we have previously shown that mtDNA variant count rises in low mtCN samples. This is indicative of potential increased NUMT contamination^4^.

All variants called by Mutect2 were emitted from mtSwirl along with filters obtained using FilterMutectCalls. During merging and annotation, several steps of variant QC were performed once sample filtration had been completed to ensure that our callset was of high quality. Briefly, the steps were as follows.

1. Any genotype with a QC filter produced by FilterMutectCalls was removed.
2. If heteroplasmy (HL) < 0.01, it was set to homoplasmic recessive.
3. If there was no genotype at a site, it was considered homoplasmic recessive provided coverage at that site was at least 100.
4. We then removed variants on the basis of heteroplasmy as follows.
  - For analysis of individual common heteroplasmic mutations, we conservatively removed any variants with heteroplasmy less than 0.05 as done previously^4^;
  - For analysis of mtSNV burden, we adopted a more lenient filter to better capture high-confidence low-heteroplasmy variation. which tends to be enriched for age accumulation (Supplementary Note 1). We identified a coverage depth for each person such that 95% of nuclear genomic sites would be expected to have less coverage under a Poisson model. We then removed SNV heteroplasmies with alternative allele depth less than this threshold.
    (i) As part of the generation of a variant callset for mtSNV burden, we also removed any variants that (in either UKB or AoU) increased by more than 500% relative to an HL > 0.05 filter and were detected more than 30 times. This resulted in exclusion of five suspicious variants: chrM:11467:A:G, chrM:12684:G,A, chrM:12705:C,T, chrM:13052:A,G and chrM:13095:T,C.

### mtCN covariate correction

Covariate correction was performed for mtCN as described previously^4^. Briefly, we obtained all blood cell composition percentages within fields 30000–30300, as well as white blood cell count (field 30,000). We (1) excluded lymphocyte percentage owing to collinearity and (2) excluded nucleated RBC percentage as most individuals had zero values. To address outliers, we removed any blood cell measurements that differed from the mean by more than four standard deviations. We also obtained several technical covariates: assessment centre, median blood draw time, fasting time, assessment date and assessment month. To model the nonlinear relationships between mtCN and draw time as well as mtCN and the seasonal effects of assessment date, we used natural splines with five degrees of freedom and seasonally placed knots (3-month increments). Assessment centre and month were modelled as indicator variables. Fasting times between 1 h and 18 h were modelled as indicators as well, with a fasting time of 0 relabelled as 1 and that greater than 18 relabelled as 18 h. All of these variables were included in a joint model:

$$\log [({\mathrm{mtCN}}_{\mathrm{raw}})] \sim \mathrm{ns}(\mathrm{blood}\,\mathrm{draw}\,\mathrm{time},\,5)+\mathrm{assessment}\,\mathrm{centre}+\mathrm{fasting}\,\mathrm{time}+\mathrm{ns}(\mathrm{assessment}\,\mathrm{date},\,\mathrm{seasonal}\,\mathrm{knots})+\mathrm{assessment}\,\mathrm{month}+\mathrm{blood}\,\mathrm{cell}\,\mathrm{variables},$$

where ns corresponds to the natural spline function. mtCN_adj_ was obtained by taking the residuals from the above model. To visualize this residual quantity, we rescaled this value by adding the preadjustment mean of log[mtCN_raw_] and then exponentiated to return to absolute scale. As aforementioned, samples obtained during the pilot study in 2006 were excluded.

### Construction of mtDNA variant classes

To visualize mtDNA variation as a function of strand, location and mutation type, we considered variants that were between positions 16,172 and 210 to be in the ‘Ori’ region, with all other variants in the ‘Other’ region. As the reference mtDNA genome was the light strand, we considered any variant with a reference allele of C or A to be a light strand variant. For variants with a reference allele of G or T, we took the complement and considered the complement allele to be a heavy strand variant. We considered ‘age-accumulating’ variant classes to comprise those variants that were in the ‘other’ region and were either A>G on either strand or C>T on the heavy strand.

### Assessment of mean mtDNA mutation count per person

We performed several analyses in which we considered the mean mtSNV count per person within subcategories of variants (for instance, variant classes or heteroplasmy categories). For these analyses, we counted the number of QC-pass SNVs identified (through the allele-depth-based filter) for each person within each subcategory. The total number of individuals included in this analysis was the number of individuals that passed QC for mtDNA variation assessment. For analyses that considered the mean count per person within subcategories of people (for instance, within age strata) we computed means within defined groups of individuals after quantifying SNV count per person as above within variant subcategories.

In cases where we compared mean SNV counts across variant classes (for instance, that shown in Fig. 2a), we normalized the estimates of mean SNV count across people by the number of possible variants in that variant class to account for differences in the base abundance across mtDNA strand or region. For instance, when comparing the mean variant count per person in the C>T other region on the light versus the heavy strand, we divided the mean observed count in the C>T other region light strand by the total number of possible C>T variants in the other region on the light strand and performed the analogous process for the C>T other region heavy strand.

For all analyses in which relationships with age were assessed, only individuals ages 18–90 years (AoU) or 40–70 years (UKB) were included to ensure sufficient samples were retained in age strata.

### Analysis of single-base substitution signatures with trinucleotide context

For each variant, trinucleotide context was obtained using the bases immediately 5′ and 3′ to the variant of interest in a strand-specific way. To perform correlations with nucDNA-based single-base substitution signatures, we obtained release v.3.4 of the COSMIC human cancer single-base signatures (SBS) collection^19^ (https://cancer.sanger.ac.uk/signatures/downloads/). For each SBS signature, a linear model was fitted to predict the observed mutational spectrum using the SBS signature. *P* values were reported from the test of significance for the beta coefficient from this model and adjusted for multiple comparisons using the Bonferroni method (two-sided *P* values, 86 total signatures).

### mtDNA variant-based phenotype construction

For analysis of individual common heteroplasmies, we identified all variants that showed QC-pass heteroplasmy (0.05 ≤ HL ≤ 0.95) in 500 or more individuals across both biobanks. This resulted in 78 common heteroplasmic variants selected for analysis. These common mtDNA variants were extracted in each of UKB and AoU, and ‘case-only’ phenotypes were constructed for each heteroplasmy by, for each heteroplasmic variant, marking any individual who failed QC or had a HL of 0 as missing and performing analysis only among those with QC-pass heteroplasmy.

For analysis of heteroplasmic SNVs, we extracted all mtSNVs passing QC with HL ≤ 0.95. We computed SNV burden by counting the number of QC-pass heteroplasmic SNVs per person, retaining people without any recorded QC-pass heteroplasmic SNVs as 0. We also computed an SNV burden trait consisting of the count of age-accumulating class variants only; this was the phenotype on which we performed the bulk of our genetic association analyses.

### Family-based analysis in UKB

Relatedness information was obtained for UKB from previous work^59^. All analyses involving related samples were performed using SNVs only. When performing transmission analysis, we only included variants that were identified in at least five individuals to ensure exclusion of artefactual variant calls. When evaluating the degree of heteroplasmic SNV sharing between siblings, we considered a variant to be shared if it was found in both siblings at any heteroplasmy. For each heteroplasmy category, we computed the proportion of variants in sibling 1 (chosen arbitrarily) that were also found in sibling 2.

### mtDNA variant consequence annotation and dN/dS estimation

Variant consequences for mtDNA mutations were inferred using VEP v.101 as implemented in Hail. For analysis of mtSNVs, we considered variants labelled as ‘synonymous_variant’, ‘stop_retained_variant’, and ‘non_coding_transcript_exon_variant’ as synonymous; ‘stop_lost’, ‘start_lost’, ‘missense_variant’ as non-synonymous, and ‘stop_gained’ as predicted loss of function.

To estimate dN/dS, we used two approaches. We generated a table of all possible mtSNVs (49,704) and used VEP v.101 to assign mutational consequence. Then, for a given region (such as a gene boundary), we estimated dN as the number of observed non-synonymous variants divided by the number of possible non-synonymous variants within the region and dS as the number of observed synonymous variants divided by the number of possible synonymous variants within the region. The ratio of these two quantities was dN/dS. For all such analyses, we restricted variants to the age-accumulating classes (C>T heavy strand and A>G both strands), as this is the mutational process we were investigating; only this subset of mutations was used for both the observed variants and the count of possible variants (that is, the denominator). In cases in which we generated a variant class- and strand-specific estimate of dN/dS, we restricted variants to the class of interest when counting both observed variants and the count of possible variants. This method was also used in the construction of per-gene dN/dS estimates for mutations found in individuals with detected CH versus in randomly sampled matched controls.

To construct a null distribution under a model of neutrality, we performed random sampling inspired by an approach described elsewhere^23^. For each gene and heteroplasmy bin, for each individual *i* and mutation class *c*, we randomly sampled ${N}_{i}^{c}$ variants from the set of all possible variants in that gene without replacement, where ${N}_{i}^{c}$ was the number of variants observed for that individual of a specific class within the gene/heteroplasmy bin. This generated a ‘callset’ under the null for which dN/dS was estimated as above. This procedure was repeated 1,000 times to generate a null distribution for dN/dS for each gene, variant class and heteroplasmy range.

As independent validation, we also used the dNdScv package^24^ to estimate dN/dS for age-accumulating variants. The dndscv command was used with default parameters, including trinucleotide and stranded context, for the mtDNA reference genome using sequence code 2. A 192-rate parameter substitution model was used. The command was run for all age-accumulating class mutations within coding regions within each heteroplasmy bin (that is, it was run separately for each heteroplasmy bin), and gene-specific dN/dS estimates were subsequently obtained from the model outputs.

The genes *MT-ATP6* and *MT-ATP8* share coding DNA sequence from chrM:8527 to chrM:8572. This meant there was a risk of introducing bias into dN/dS estimates, as mutations within this region face selection forces from two different genes and codon reading frames. Thus, for all dN/dS estimation methods, we corrected for sequence sharing between *MT-ATP6* and *MT-ATP8* by excluding this region from all stages of the analysis.

### UKB disease and smoking phenotype curation

To assess the association of mtDNA phenotypes and common diseases in UKB, we used a previously curated set of 28 common disease phenotypes based on ICD-10 codes, categorical traits curated by UKB and phecodes that spanned several different disease domains^60^. To simplify the cancer terms included in this phenotype set, we constructed two new ICD-10-based traits: haematologic cancer, which involved a diagnosis of C81–C96 or D45–D47; and solid tumour, which involved a diagnosis of C00–C75.

To further evaluate the associations between mtSNV burden and cancer-related phenotypes, we extracted all phecodes with the category of ‘neoplasm’ as curated in previous work^60^. We manually excluded traits corresponding to benign conditions (such as lipoma) or otherwise non-specific traits (for instance, malignant neoplasm other). We filtered to only phenotypes that had more than 140 observed cases in UKB. We obtained smoking status from UKB by obtaining the ID 20116.

### Correlation of mtCN and SNV count with disease traits

We computed odds ratios for the associations between mtDNA traits (mtCN, SNV burden) and categorical disease traits using logistic regression as implemented in R in UKB. For all mtDNA traits and disease phenotypes we included genetic ancestry assignment, age, age × sex, age^2^ and age^2^ × sex as covariates, as well as haplogroups as indicator variables. Genetic ancestry groups with fewer than three individuals with any given defined mtDNA trait or disease phenotype were excluded. For analyses in which the independent variable was mtDNA heteroplasmic SNV burden, only age-accumulating variants were used in the burden metric. Individuals who were described as ever having smoked were excluded for all analyses with mtSNV burden.

### Correlation of mtCN and SNV count with protein levels and telomere length

We assessed the correlations of mtCN and mtSNV burden with levels of several proteins (FGF21, GDF15, IL1A, IL6, TNF) and telomere length. Proteomics data were obtained as generated and processed by the UKB Pharma Proteomics Project team^61^. In brief, UKB plasma samples were obtained and processed through Olink Explore 3072, which provides a platform capable of targeting 2,923 unique proteins. Results were normalized to sample- and plate-specific controls and then corrected for batch. More information can be found using resources 4654–4658 in the UKB Showcase. Field 30900 from UKB contains the normalized proteomics data. For this study, proteomics data were obtained from the 50k release, which was a random subsample of the total UKB population. In terms of further postprocessing, missing data were imputed using the miceforest package as described previously^62^. Telomere length data were also obtained from UKB from field 22192 (‘Z-adjusted T/S log’). These measurements were obtained as described previously^63^. In brief, DNA from virtually the entire UKB cohort was assayed for telomere length at the University of Leicester by using robot-assisted multiplex qPCR to measure the ratio of telomere repeat copy number relative to a single copy gene. Extensive QC was performed, including removal of samples with a high coefficient of variation and abnormal PCR melt curves. Raw telomere-to-single copy gene ratios were adjusted for several technical factors including batch, machine, temperature, time of day and time-to-collection. Samples were then Z-standardized and log-transformed. As suggested in previous literature^63^, we adjusted telomere length for white blood cell count using a linear model. This adjusted telomere length was subsequently used for analysis.

Associations between processed telomere length and protein phenotypes and mtCN and mtSNV burden were computed similarly to those done for disease traits. Namely, a linear regression model was used to identify the effect size of changes in mtCN or mtSNV count with respect to telomere length and protein levels while correcting for genetic ancestry assignment, age, age × sex, age^2^, age^2^ × sex and haplogroup. For associations with mtSNV count, we restricted analysis to never-smokers.

### Variant-based testing and meta-analysis

In UKB, we performed GWAS using a similar approach to that described previously^4,60^. In brief, GWAS was run on GRCh37-based imputed array data using SAIGE v.1.3.6^64^ within previously defined genetic ancestry groups^60^. SAIGE was run using a full genetic relatedness matrix (GRM) in the null model step with leave-one-chromosome-out enabled. All phenotypes were inverse-rank normalized before genetic analysis.

In AoU, we performed GWAS on GRCh38-based WGS data using SAIGE v.1.4.3. We performed extensive sample and variant QC before analysis, in an approach similar to that used by the AoU All-by-All initiative (see https://support.researchallofus.org/hc/en-us/articles/27049847988884-Overview-of-the-All-by-All-tables-available-on-the-All-of-Us-Researcher-Workbench for more details). Unimputed genotype data and WES and WGS data were all used in the generation of files to run SAIGE. Across all data modalities, sample and variant QC were performed as follows. For sample QC, genetic ancestry assignment and ancestry outliers for removal were obtained from the All-by-All effort. Samples were also removed if they were flagged by the AoU DRC. For variant QC, variants were removed if they failed ‘adj’ criteria^59^ or if their ancestry-specific call rate was less than 90%. Ancestry-specific principal components were computed after sample QC on unrelated individuals with projection of related individuals on to the computed principal component space.

To generate the sparse GRM in AoU for each ancestry group, we performed linkage disequilibrium pruning using PLINK on post-QC autosomal genotype data. Linkage disequilibrium pruning was performed using a step size of 1, window size of 10,000 kb, and r2 of 0.1. We excluded the HLA locus and the chromosome 8 inversion locus. Linkage-disequilibrium-pruned genotype data were then exported to PLINK format, and sparse GRMs were constructed within each ancestry with 2,000 markers, a minimum allele frequency of 0.01 and a relatedness cutoff of 0.125 using SAIGE.

Next, to generate files relevant for generation of null models in AoU, we subsampled both WGS and WES data. To ensure that we captured the full range of allele frequencies (given that WGS data were only provided for variants that had allele frequency > 0.01 in at least one ancestry group, that is, ACAF WGS), we obtained 50,000 common autosomal variants at random (allele frequency > 0.01) from WGS data and appended randomly sampled rare variants from WES data. Rare variants were sampled within the following categories: minor allele count (MAC) 1, 2, 3, 4, 5, 6–10, 11–20; minor allele frequency (MAF) < 0.001, MAF < 0.01 and MAF > 0.01. Two-thousand variants were extracted from each MAC category and 10,000 variants were obtained from each MAF category. This was done separately for each population. Linkage disequilibrium pruning was then performed on the combined table of subsampled rare and common variants after removal of HLA and the chromosome 8 inversion locus using r2 0.1 and a window size of 10,000 kb. The resulting pruned variants were exported to PLINK format.

Finally, to efficiently run SAIGE tests, we obtained QC-pass WGS data and then exported BGEN files tiled across the genome separately for each population. This greatly increased parallelism. In AoU, as SAIGE was run with a sparse GRM for improved computational efficiency, leave-one-chromosome-out was disabled.

In both biobanks, all GWAS included ‘baseline’ covariates including the ancestry-specific principal components (10 in UKB, 20 in AoU), age, age × sex, age^2^, and age^2^ × sex. Ancestry groups were only included in the analysis if the analysis had a sample size of 50 or greater. For all mtDNA variant-based analyses (that is, heteroplasmy and SNV burden), we included indicator variables for top-level haplogroup assignments obtained from mtSwirl. We removed any individuals belonging to haplogroups that had fewer than 30 people in either biobank; this resulted in removal of individuals from haplogroups L5, P, Q and S. These were not included for analyses of mtCN. In AoU, a further covariate corresponding to the sequencing site was included. GWAS was always run on phenotypes after inverse-rank normal transformation. Covariate offset was disabled.

For mtDNA heteroplasmy GWAS, we ran analyses using both additive and recessive encoding. In the case of the recessive encoding, BGEN files were re-exported in both biobanks after recoding of heterozygous individuals as homozygous reference. No changes were made in inputs to the null model step.

When GWAS were complete, we performed summary statistics QC. This involved removal of any low-confidence variants (defined as MAC < 20 or call rate < 0.9) and dropping of phenotypes with lambda GC < 1.5. We then performed fixed-effects meta-analysis with inverse-variance weighting within each cohort to meta-analyse populations, followed by another round of fixed-effects meta-analysis with inverse-variance weighting across cohorts. As UKB GWAS was performed in GRCh37, we used a previously generated liftover file to transfer UKB variants to GRCh38 for meta-analysis^4^.

### Fine-mapping of UKB GWAS

To identify putative causal variants, we conducted statistical fine-mapping of mtDNA traits in UKB as previously described^4,60,65^. Briefly, we used FINEMAP-inf and SuSiE-inf, which model infinitesimal effects^66^, with cross-ancestry meta-analysis summary statistics from UKB and a covariate-adjusted in-sample dosage linkage disequilibrium matrix. To reduce computational burden due to the increased number of genome-wide significant loci, we defined fine-mapping regions on the basis of a 1-Mb window (rather than the previously described 3 Mb^4,60,65^) around each lead variant and merged overlapping regions. This resulted in 802 genome-wide significant regions for fine-mapping, of which only 5 loci exceeded 3 Mb, with a maximum length of 5.2 Mb. This approach properly handles long-range linkage disequilibrium regions^67^ while keeping fine-mapping region sizes manageable. We excluded the major histocompatibility complex region (chr6: 25–36 Mb) from analysis, owing to its extensive linkage disequilibrium structure. Allowing up to ten causal variants per region, we derived PIPs using each method with a uniform prior probability of causality and computed the minimum PIP across methods. For 95% credible sets, we used those reported by SuSiE-inf.

### Identification of loci and assignment of genes

Owing to the multiancestry and multibiobank nature of our cohort, we used a proximity-based approach to identify separate loci. Specifically, for each GWAS, we identified initial SNVs with *P* < 5 × 10^−5^ and then constructed a window of 100 kb up and down from each such variant. We then iterated through variants in order and merged variants with overlapping windows. A locus was defined as a merged set of variants non-overlapping with any other set, and lead nucDNA variants for a given locus were identified by that with the most significant *P* value. This process was repeated for published GWAS for CH to obtain lead variants.

For gene assignment, where fine-mapped variants were identified with PIP > 0.1, gene assignment was performed by a combination of nearest gene and manual curation in the genomic region proximal to the fine-mapped variant. Genes in which a protein coding change was predicted by the fine-mapped variant were prioritized. If no fine-mapped variants with PIP > 0.1 were detected, a combination of nearest gene and manual curation was used.

### One-sided Mendelian randomization between CH and mtSNV burden

Summary statistics from a recent well-powered case-control analysis of aggregate CH were obtained (GWAS catalogue ID GCST90165261)^2^. Location-based lead variant identification was performed as described above. Odds ratios for lead variants were transformed to log-odds scale, and allele direction was set such that all effect sizes were risk-increasing. Effect sizes and standard errors for these variants from heteroplasmic mtSNV count GWAS were then obtained, ensuring that allele direction was concordant. To determine the statistical significance of the correlation between effect sizes, inverse-variance weighted linear regression was used with weights given by $1/S{E}_{x}^{2}\times 1/S{E}_{y}^{2}$.

### Gene-based testing and meta-analysis

We performed gene-based testing in UKB using the OQFE 500k whole-exome data release. Owing to limited sample sizes for non-European ancestry assigned groups, we performed our analyses using the 500k exome data from individuals of European ancestry only. We used SAIGE-GENE+^64^ v.1.4.3 with identical covariates and phenotypes to those used for variant-based GWAS in UKB. To produce null models, we extracted genotyped variants in the following categories: MAC 1, 2, 3, 4, 5, 6–10, 11–20; MAF < 0.001, MAF < 0.01 and MAF > 0.01. Two-thousand variants were extracted from each MAC category, and 10,000 variants were obtained from each MAF category at random. PLINK was used to perform linkage disequilibrium pruning to obtain a linkage-disequilibrium-independent set of variants for variance ratio estimation. Variance ratios were estimated separately by MAC category, given by MAC 1, 2, 3, 4, 5, 6–10, 11–15, 16–20 and 21+. Assignments of variants to genes and consequence annotations were obtained from previous work^64^.

To perform gene-based testing in AoU, we used the same approaches for variant and sample QC as for variant-based testing. The same sparse GRM was used as well. Analyses were performed in all six ancestry groups. To generate the variant annotation group file, we used the variant annotation table provided by AoU (v.7.1). For each gene, the worst consequence across all transcripts was used to assign each variant to either synonymous, missense or predicted loss of function. Variants with ancestry-wide call rate less than 0.9 were excluded.

To generate null models for rare-variant analysis in AoU, the same input genotypes were used as for common variant analysis, namely a subsampled set of variants with AF > 0.01 using ACAF WGS data combined with subsampled variants with MAC 1, 2, 3, 4, 5, 6–10, 11–20; MAF < 0.001, MAF < 0.01, MAF > 0.01. SAIGE was run with the same parameters as for AoU common variant analysis except with the ‘—isCateVarianceRatio’ option enabled, generating variance ratio estimates for MAC 1, 2, 3, 4, 5, 6–10, 11–15, 16–20 and 21+. These were the same categories used for gene-based testing in UKB.

For parallelism in AoU, we generated BGEN files comprising QC-pass WES-based variant calls tiled across the genome for each population. We adjusted the cut points of these tiles to ensure that gene boundaries were not split across multiple files. These files were used to run SAIGE tests. In AoU, as SAIGE was run with a sparse GRM for improved computational efficiency, leave-one-chromosome-out was disabled.

In both biobanks, a total of 15 tests were performed per gene: maximum MAF values of 0.01, 0.001, and 0.0001; missense/LoF/synonymous, missense/LoF, LoF, missense and synonymous annotations; and a Cauchy combination test combining evidence across annotations. SKAT-O^68^ was primarily used for assessment of gene-based associations. To minimize comparisons, we used the Cauchy test to nominate phenotypes for further evaluation and then evaluated associated variant groups only for those phenotypes. Our genome-wide threshold was set using the Bonferroni method with a threshold of 0.05 divided by ~18,000 corresponding to the number of tested genes, and our ‘suggestive’ threshold was a false discovery rate of 0.1 using the Benjamini–Hochberg procedure.

To conduct cross-ancestry and cross-biobank meta-analysis of gene-based tests, we used both inverse-variance weighted meta-analysis and Stouffer’s weighted *P* value combination method. Inverse-variance weighting was used to combine burden tests to produce effect size estimates. Stouffer’s weighted *P* value combination method^69^ was used to combine Burden, SKAT, SKAT-O and Cauchy *P* values. Weights were constructed as $\sqrt{N}$, where *N* is the sample size of a given ancestry or biobank. This meta-analysis procedure was first performed in AoU to combine per-ancestry results and then repeated to combine UKB summary statistics with AoU summary statistics.

### Identification of individuals with CH in UKB and AoU

Individuals with CH were obtained from both UKB and AoU as described in detail elsewhere^70^. In brief, CH was identified by assessing for putative somatic variants in WGS data within a curated list of known driver genes for CH by identifying fractional variants with Mutect2, followed by extensive QC and removal of suspected germline variants.

### Age- and sex-matching in analyses of individuals with CH

CH is known to be strongly associated with age^70^. Given this, in analyses comparing individuals with detected CH with those without detected CH, we performed age- and sex- matching to minimize the risk of confounding by these variables. We obtained ages and sexes of individuals with known CH and then constructed 500 random samples of people without diagnosed CH of the same size as the CH group. Each of these random samples consisted of people with the same sex and age counts as the CH group. We then computed statistics within each of these control groups (for instance, mean variant count per person within a variant class and age group) and then computed the mean across all random subsamples as the point estimate and the standard deviation of the means across subsamples as the standard error.

For the analysis comparing the normalized mean mutation count between CH and age- and sex-matched controls, we also computed a difference statistic within each random sample; this was the difference in the mean mutation count for a mutation in a particular trinucleotide context between the CH sample and then random sample. We then obtained the point estimate and standard error of the difference statistic using the mean and standard deviation of the difference statistic across all 500 samples, respectively. Significance tests were performed with a null hypothesis of difference of 0 and a two-sided alternative of difference not equal to 0, and *P* values were corrected using the Bonferroni method assuming 192 comparisons (96 trinucleotide contexts × 2 strands).

### Reporting summary

Further information on research design is available in the Nature Portfolio Reporting Summary linked to this article.

## Online content

Any methods, additional references, Nature Portfolio reporting summaries, source data, extended data, supplementary information, acknowledgements, peer review information; details of author contributions and competing interests; and statements of data and code availability are available at 10.1038/s41586-026-10569-6.

## Supplementary information

Supplementary Notes Supplementary Notes 1–3, including Supplementary Figs. 1–5 and additional references.

Reporting Summary

Supplementary Tables Supplementary Tables 1–12, containing sample sizes, lead variants, significant gene-based association test results, and fine-mapping results from GWAS/RVAS of all traits tested in this study.

Peer Review File

## Data Availability

The source data analysed in this study were obtained from UKB and AoU. The UKB data were accessed under application 31063. UKB WGS data were accessed and analysed within the UKB Researcher Analysis Platform (https://ukbiobank.dnanexus.com/landing). AoU data were accessed as part of three custom workspaces: ‘Genetic determinants of mitochondrial DNA phenotypes (v7)’, ‘Genetic determinants of mitochondrial DNA phenotypes (v7) second 40k’ and ‘Genetic determinants of mitochondrial DNA phenotypes (v7) finish pipeline’. All analysis of AoU data was performed within the AoU Researcher Workbench (https://workbench.researchallofus.org/) using the workspaces described above. Summary statistics from mtCN and heteroplasmy GWAS were obtained from previous work^4^. Summary statistics from CH GWAS were obtained from work by Kessler et al.^2^ (GWAS catalogue accession GCST90165261). GRCh37 and GRCh38 reference genomes and other reference sequence-related data were obtained from the GATK resource bundle (https://gatk.broadinstitute.org/hc/en-us/articles/360035890811-Resource-bundle). mtDNA local constraint scores were obtained from work by Lake et al.^37^. The previously curated set of common disease traits in UKB was obtained from previous work^60^; more information can be found online at https://pan.ukbb.broadinstitute.org. COSMIC SBS signatures were obtained from https://cancer.sanger.ac.uk/signatures/downloads/. Sample sizes for cross-biobank meta-analysis of heteroplasmy GWAS and SNV count GWAS are provided in Supplementary Tables 1 and 2 respectively. Lead SNPs from proximity-based clumping at a *P* value threshold of 5 × 10^−5^ for mtCN_adj_, heteroplasmy GWAS and SNV count GWAS are included in Supplementary Tables 3, 4 and 5 respectively. Gene-based test results for SNV count are included for genes with *P* < 5 × 10^−5^ in Supplementary Table 6. All variants with fine-mapped PIP > 0.1 in any tested trait in UKB are included in Supplementary Table 7. Raw effect sizes and *P* values from tests of association between mtDNA traits and disease are available in Supplementary Tables 8–10. Association *P* values between CH and mutation levels in various mutation classes are provided in Supplementary Tables 11 and 12. All of our largest sample size GWAS summary statistics (cross-biobank meta-analysis when possible, single biobank otherwise) are available on GWAS Catalog (study accession GCP001670). We have made AoU calls and full meta-analysis summary statistics available on the AoU Workbench under a workspace titled ‘Mechanism of age-related accumulation of mtDNA mutations in human blood’. We have made UKB meta-analysis summary statistics available on Google Cloud Platform (see GitHub repository in Code Availability). We are in the process of returning our mtDNA variant and copy number calls to UKB for broader use.

## Code availability

All code related to the updated version of mtSwirl (v.2) and to generate the callset is available at on GitHub (https://github.com/rahulg603/mtSwirl). Software versions used within the mtSwirl pipeline have not changed and can be found elsewhere^4^. This includes GATK v.4.2.6.0 (same as previously), which includes Mutect2. The mtSwirl repository includes code used to perform GWAS in UKB. Code written to generate phenotypes for downstream analysis in AoU, perform GWAS and/or RVAS in AoU, and perform meta-analysis (cross-ancestry and cross-biobank) can also be found at GitHub (https://github.com/rahulg603/mtdna_750k_gwas). To perform downstream analyses, Hail was used for parallelized analyses (v.0.2.120 in UKB and v.0.2.130-post1 in AoU; v.0.2.119 used in pipelines), with single-machine data processing, statistical testing and figure generation done using R v.4.4.0 (most analysis) and v.4.2.1 (munging of UKB phenotypes). Tasks were parallelized using Hail Batch in UKB (https://hail.is/docs/batch/index.html) and Cromwell in AoU (https://cromwell.readthedocs.io). Linkage disequilibrium pruning was performed using PLINK v.1.9 (https://www.cog-genomics.org/plink/). GWAS were performed using SAIGE v.1.3.6 (UKB GWAS) or v.1.4.3 (UKB RVAS and AoU analyses) (https://saigegit.github.io/SAIGE-doc/). As part of our analyses, we used VEP v.101 as implemented in Hail to assign consequence to mtDNA variation. dndscv v.0.0.1.0 was used to quantify dN/dS in mtDNA. Plots were generated using ggplot2 v.3.5.2; rounded rectangle annotations were generated using ggchicklet v.0.5.2, and certain combined plots were generated using cowplot v.1.1.3.
