# Supplementary material for: Mechanism of age-related accumulation of mtDNA mutations in human blood
Source: Nature. 2026 May 27;656(8126):140–8. doi: 10.1038/s41586-026-10569-6 (PMC13441879; doi:10.1038/s41586-026-10569-6)
Supplement: Supplementary file 2 [file 41586_2026_10569_MOESM2_ESM.pdf]

nature portfolio

Corresponding author(s): Vamsi K. Mootha, Benjamin M. Neale
Rahul Gupta (submitting)

Last updated by author(s): Apr 5, 2026

Reporting Summary

Nature Portfolio wishes to improve the reproducibility of the work that we publish. This form provides structure for consistency and transparency in reporting. For further information on Nature Portfolio policies, see our Editorial Policies and the Editorial Policy Checklist.

Statistics

For all statistical analyses, confirm that the following items are present in the figure legend, table legend, main text, or Methods section.

n/a	Confirmed	
☐	☒	The exact sample size (*n*) for each experimental group/condition, given as a discrete number and unit of measurement
☒	☐	A statement on whether measurements were taken from distinct samples or whether the same sample was measured repeatedly
☐	☒	The statistical test(s) used AND whether they are one- or two-sided<br>*Only common tests should be described solely by name; describe more complex techniques in the Methods section.*
☐	☒	A description of all covariates tested
☐	☒	A description of any assumptions or corrections, such as tests of normality and adjustment for multiple comparisons
☐	☒	A full description of the statistical parameters including central tendency (e.g. means) or other basic estimates (e.g. regression coefficient) AND variation (e.g. standard deviation) or associated estimates of uncertainty (e.g. confidence intervals)
☐	☒	For null hypothesis testing, the test statistic (e.g. *F*, *t*, *r*) with confidence intervals, effect sizes, degrees of freedom and *P* value noted<br>*Give P values as exact values whenever suitable.*
☒	☐	For Bayesian analysis, information on the choice of priors and Markov chain Monte Carlo settings
☒	☐	For hierarchical and complex designs, identification of the appropriate level for tests and full reporting of outcomes
☒	☐	Estimates of effect sizes (e.g. Cohen's *d*, Pearson's *r*), indicating how they were calculated

Our web collection on statistics for biologists contains articles on many of the points above.

Software and code

Policy information about availability of computer code

Data collection	No data was collected in this study as we analyzed existing data.
Data analysis	All code related to the updated version of mtSwirl (v2) and to generate the callset can be found on Github (https://github.com/rahulg603/mtSwirl). Software versions used within the mtSwirl pipeline have not changed and can be found elsewhere (Gupta et al. 2023). Importantly, this includes GATK v4.2.6.0 (same as prior), which includes Mutect2. The mtSwirl repository includes code used to perform GWAS in UKB. Code written to generate phenotypes for downstream analysis in AoU, perform GWAS/RVAS in AoU, and perform meta-analysis (cross-ancestry and cross-biobank) can be found on Github as well (https://github.com/rahulg603/mtdna_750k_gwas).<br><br>To perform downstream analyses, Hail was used for parallelized analyses (v0.2.120 in UKB and v0.2.130-post1 in AoU; v0.2.119 used in pipelines), with single-machine data processing, statistical testing, and figure generation done using R v4.4.0 (most analysis) and v4.2.1 (munging of UKB phenotypes). Parallelization of tasks was achieved using Hail Batch in UKB (https://hail.is/docs/batch/index.html) and using the inbuilt AoU Cromwell Server (https://cromwell.readthedocs.io). LD-pruning was performed using PLINK v1.9 (https://www.cog-genomics.org/plink/). GWAS was performed using SAIGE v1.3.6 (UKB GWAS) or v1.4.3 (UKB RVAS and AoU analyses) (https://saigegit.github.io/SAIGE-doc/). As part of our analyses, we used VEP v101 as implemented in Hail to assign consequence to mtDNA variation. dndscv v0.0.1.0 was used to quantify dN/dS in mtDNA. Plots were generated using ggplot2 v3.5.2. Rounded rectangle annotations in plots were generated using ggchicklet v0.5.2. Certain combined plots were generated using cowplot v1.1.3.

For manuscripts utilizing custom algorithms or software that are central to the research but not yet described in published literature, software must be made available to editors and reviewers. We strongly encourage code deposition in a community repository (e.g. GitHub). See the Nature Portfolio guidelines for submitting code & software for further information.

Data

Policy information about availability of data

All manuscripts must include a data availability statement. This statement should provide the following information, where applicable:

- Accession codes, unique identifiers, or web links for publicly available datasets
- A description of any restrictions on data availability
- For clinical datasets or third party data, please ensure that the statement adheres to our policy

In terms of external data used in this study, the source data analyzed in this study was obtained from UKB and AoU. The data was accessed in UKB under application 31063. UKB WGS data was accessed and analyzed within the UKB Researcher Analysis Platform (https://ukbiobank.dnanexus.com/landing). AoU data was accessed as part of three custom workspaces: “Genetic determinants of mitochondrial DNA phenotypes (v7)”, “Genetic determinants of mitochondrial DNA phenotypes (v7) second 40k”, and “Genetic determinants of mitochondrial DNA phenotypes (v7) finish pipeline”. All analysis of AoU data was performed within the AoU Researcher Workbench (https://workbench.researchallofus.org/) using the workspaces described above. Summary statistics from mtDNA copy number and heteroplasmy GWAS were obtained from prior work (Gupta et al. 2023). Summary statistics from CH GWAS were obtained from work by Kessler et al. 2022 (GWAS Catalog accession GCST90165261). GRCh37 and GRCh38 reference genomes and other reference sequence-related data were obtained from the GATK resource bundle: https://gatk.broadinstitute.org/hc/en-us/articles/360035890811-Resource-bundle. MLC scores were obtained from recent work by Lake et al. 2024. The previously curated set of common disease traits in UKB was obtained from prior work54; more information can be found online: https://pan.ukbb.broadinstitute.org. COSMIC SBS signatures were obtained from: https://cancer.sanger.ac.uk/signatures/downloads/

We include sample sizes for cross-biobank meta-analysis of heteroplasmy GWAS and SNV count GWAS in Supplementary Tables 1 and 2 respectively. Lead SNPs from proximity-based clumping at a p-value threshold of 5e-5 for mtCNadj, heteroplasmy GWAS, and SNV count GWAS are included in Supplementary Tables 3, 4, and 5 respectively. Gene-based test results for SNV count are included for genes with p-value < 5e-5 in Supplementary Table 6. All variants with fine-mapped posterior inclusion probability (PIP) > 0.1 in any tested trait in UKB are included in Supplementary Table 7. Raw effect sizes and p-values from tests of association between mtDNA traits and disease are available in Supplementary Tables 8, 9, and 10. Association p-values between CH and mutation levels in various mutation classes are released in Supplementary Tables 11 and 12. We have made AoU calls and full meta-analysis summary statistics available on the AoU Workbench under the workspace titled “Mechanism of age-related accumulation of mtDNA mutations in human blood”. We are in the process of returning our mtDNA variant and copy number calls to UKB for broader use.

Research involving human participants, their data, or biological material

Policy information about studies with human participants or human data. See also policy information about sex, gender (identity/presentation), and sexual orientation and race, ethnicity and racism.

Reporting on sex and gender	We use existing cohorts and do not perform any recruitment in this study. We obtain information on sex via the corresponding variable in UKB or via genetic inference in AoU. We use sex as a covariate for many analyses performed in this paper as this can be an important confounder.
Reporting on race, ethnicity, or other socially relevant groupings	We use existing cohorts for the analyses performed in this paper. We obtain genetic ancestry assignments from prior efforts (Karczewski et al. 2024 medRxiv for UKB, and via the AoU Data and Research Center for AoU). To avoid population stratification and associated confounding we perform our GWAS/RVAS within each genetic ancestry assignment and then perform meta-analysis. Genetic ancestry is also used as a covariate in several of our analyses correlating mtDNA to phenotypes. This manuscript does not use race or SES or other socially relevant groupings.
Population characteristics	We did not perform any recruitment in this study. We filtered on age ranges to avoid outlier effects: ages 40-70 in UKB and ages 18-90 in AoU, when age was a relevant variable in our analysis. Otherwise, we did not filter our data on any population level characteristics.
Recruitment	We did not perform new recruitment for this study. For more information on the recruitment procedures of UKB and AoU see: UK Biobank – Sudlow et al. 2015 PLOS Medicine; UK Biobank WGS – Halldorsson et al., 2022 Nature; AllofUs – “The ‘All of Us’ Research Program.”
Ethics oversight	We used existing cohorts and datasets. We accessed UKB data within guidelines under application 31063. We accessed AoU data under the v7 controlled tier authorization under the following workspaces: “Genetic determinants of mitochondrial DNA phenotypes (v7)”, “Genetic determinants of mitochondrial DNA phenotypes (v7) second 40k”, and “Genetic determinants of mitochondrial DNA phenotypes (v7) finish pipeline”. An IRB was not required for this study.

Note that full information on the approval of the study protocol must also be provided in the manuscript.

Field-specific reporting

Please select the one below that is the best fit for your research. If you are not sure, read the appropriate sections before making your selection.

☒ Life sciences ☐ Behavioural & social sciences ☐ Ecological, evolutionary & environmental sciences

For a reference copy of the document with all sections, see nature.com/documents/nr-reporting-summary-flat.pdf

Life sciences study design

All studies must disclose on these points even when the disclosure is negative.

Sample size	In this study we performed the largest possible analysis using existing WGS data in AoU and UKB. No new recruitment was performed, and as such no sample size calculation was performed. This is currently the largest analysis of WGS-based mtDNA calls to date.
Data exclusions	We performed our analysis on biobank-scale data. For all analyses we excluded samples with evidence of contamination or that had "overlapping homoplasmies" which was an indication of failure in the mtSwirl pipeline. For analysis of mtDNA variation, we additionally excluded samples with mtCN < 50 as these samples have been shown to have a higher risk of NUMT contamination. We also excluded UKB samples processed in 2006 as these had abnormal mtCN, and also removed AoU samples failing QC as recommended by AoU DRC: https://support.researchallofus.org/hc/en-us/articles/27633757470228-All-of-Us-Genomic-Quality-Report-Archived-C2022Q4R13-CDRv7. These were pre-established exclusions and have been used in prior work (Gupta et al. 2023 Nature, Laricchia et al. 2022 Genome Res). We additionally excluded samples which were part of ancestry groups that were too rare for interpretability – see Methods. For analysis of phenotypes with mtDNA SNV burden, we excluded individuals with a history of smoking given the probable confounding role it has by impacting both mtDNA SNV burden and cancer risk.
Replication	We had several approaches to verify reproducibility of our experimental findings. First, we compared our mtCN results to prior work in UKB and found replication of nearly all associations. Specifically, all associations found in this study were compared to prior association statistics in UKB. Second, for all analyses collating the mutational spectrum of mtDNA, the degree of selection, and the effects of clonal hematopoiesis on mtDNA SNV burden, we performed the analyses separately in both biobanks and found highly concordant results. Third, for GWAS and RVAS of SNV burden, we compared the genetic architecture between the two biobanks and found that they were highly similar, once again showcasing the robustness of our results. We then performed meta-analysis to improve our power. Finally, we compared results between gene-based rare variant and variant-based common variant analyses and found that they had significant overlap. Specifically, for mtDNA copy number and mtSNV burden, rare variant testing results were compared to GWAS results and concordance was seen for several hits. This was accomplished via inspection.
Randomization	We did not have an experimental arm in this study, as we focused on analysis of existing biobank-scale data. We performed extensive covariate control. For all GWAS/RVAS we performed analysis within ancestry groups so as to minimize the risk of population stratification. We additionally corrected for the first 10-20 genetic principal components (computed within genetic ancestry group) to further reduce the risk of stratification. We corrected for several other standard covariates including age, sex, age2, age2*sex, age*sex. We corrected for haplogroup as well when performing GWAS/RVAS of heteroplasmy or SNV count. In AoU, we additionally controlled for the site of sequencing. For analyses of phenotypes, we included several of the above covariates including genetic ancestry, age, sex, age2, age2*sex, age*sex. For the analysis of mtCN, as described previously in Gupta et al. 2023 Nature, we corrected for a host of confounders including blood cell composition, blood draw time, seasonality, and more. See Methods.
Blinding	Blinding was not relevant for this study as there was no experimental arm of this study.

Reporting for specific materials, systems and methods

We require information from authors about some types of materials, experimental systems and methods used in many studies. Here, indicate whether each material, system or method listed is relevant to your study. If you are not sure if a list item applies to your research, read the appropriate section before selecting a response.

Materials & experimental systems

n/a	Involved in the study
☒	☐ Antibodies
☒	☐ Eukaryotic cell lines
☒	☐ Palaeontology and archaeology
☒	☐ Animals and other organisms
☒	☐ Clinical data
☒	☐ Dual use research of concern
☒	☐ Plants

Methods

n/a	Involved in the study
☒	☐ ChIP-seq
☒	☐ Flow cytometry
☒	☐ MRI-based neuroimaging

Plants

Seed stocks

Report on the source of all seed stocks or other plant material used. If applicable, state the seed stock centre and catalogue number. If plant specimens were collected from the field, describe the collection location, date and sampling procedures.

Novel plant genotypes

Describe the methods by which all novel plant genotypes were produced. This includes those generated by transgenic approaches, gene editing, chemical/radiation-based mutagenesis and hybridization. For transgenic lines, describe the transformation method, the number of independent lines analyzed and the generation upon which experiments were performed. For gene-edited lines, describe the editor used, the endogenous sequence targeted for editing, the targeting guide RNA sequence (if applicable) and how the editor was applied.

Authentication

Describe any authentication procedures for each seed stock used or novel genotype generated. Describe any experiments used to assess the effect of a mutation and, where applicable, how potential secondary effects (e.g. second site T-DNA insertions, mosiacism, off-target gene editing) were examined.
