# Supplementary material for: Mechanism of age-related accumulation of mtDNA mutations in human blood
Source: Nature. 2026 May 27;656(8126):140–8. doi: 10.1038/s41586-026-10569-6 (PMC13441879; doi:10.1038/s41586-026-10569-6)
Supplement: Supplementary file 3 [file 41586_2026_10569_MOESM4_ESM.pdf]

nature portfolio

Peer Review File

Mechanism of age-related accumulation of mtDNA mutations in human blood

Corresponding Author: Dr Vamsi Mootha

Any redactions in this file are there to maintain patient confidentiality, the confidentiality of unpublished data, or to remove third-party material.

This file contains all reviewer reports in order by version, followed by all author rebuttals in order by version.

Version 1:

Reviewer comments:

Referee #1

(Remarks to the Author)
In this manuscript, Gupta and colleagues build on their previous work and address a long-standing question in the field of mitochondrial biology: Why does the mitochondrial genome accumulates mutations as we age? To do so, the authors analyze WGS data from >700K participants from the AoU and UKBB cohorts. In the first part of the manuscript, they assessed the quality of their new mitochondrial genomic dataset by extending many analyses performed in their earlier study. Using GWAS, they replicated and identified many loci associated with mitochondrial copy number and mtDNA variant heteroplasmy, highlighting many strong candidate genes. Subsequent analyses are more interesting and novel. They suggested that age-accumulating variants are unlikely due to oxidative stress, probably somatic in nature, and not under positive selection. One of the most insightful observations came from a GWAS of mtDNA SNV burden, which identified several loci also associated with clonal hematopoiesis (CH). This and other observations led the authors to propose a model by which heteroplasmy increases during aging as a consequence of the proliferative advantage confers by CH driver somatic mutations.

The strengths of the study include the very large sample size, the consistency of the results between AoU and UKBB, a strong analytical plan and a well-written manuscript. While the first part of the paper is an extension of the previous work, it serves to validate the call set. The molecular model (CH leading to increase heteroplasmy) is plausible and well-supported by the data. However, it does raise the question of the clinical (causal) relevance of accumulating heteroplasmic variants in aging and diseases. I have only a few comments:

1. Do the main results indicate that age-related accumulation of mtDNA SNV is not particularly important for human health, but rather a bystander product of CH? Can the authors extend on this in the Discussion? What are the implications of these results for mitochondrial diseases? How should researchers working in this field consider mtDNA SNV? Should they be ignored? Should the focus be only on mtDNA indels? The study is important and will impact the field, so guidance should be provided.

2. What is known about the impact of mtDNA mutations on mitochondrial functions? I guess that somatic heteroplasmy variants have little/no impact (based on the selection analyses). What about the maternally-inherited variants?

3. The suggestion that mtDNA SNV burden could be earlier biomarkers of CH is interesting, and could be tested. For instance, after removing known CH carriers, is the mtDNA SNV burden still associated with hematologic cancers?

4. Potentially using the UKBB proteomic data and excluding the known CH carriers, do participants with a high mtDNA SNV burden have higher levels of pro-inflammatory cytokines (e.g. IL6, TNFa), as observed in CH carriers (after correcting for age)?

5. Mediation analyses. Does the accumulation of mtDNA SNV mediate the effect of CH on blood cancer risk? I would think the answer is no, but it would be nice to show.

6. Along the same line, I think that Fig.3B captures the MR analysis performed by the authors, but it is not explicitly described in the text (there is one paragraph in the Methods). Note that bi-directional MR would be informative to re-enforce the model that CH causes age-accumulating mtDNA SNV (and not the other way around).

7. The authors analyzed genetically diverse cohorts but did not detailed how many participants from each genetically-defined groups were considered (or I missed it). Were they ancestry-specific significant GWAS findings (which would not simply be due to power)?

8. (Minor). Fig. 2C. Add scale on x-axis. Same for Ext. Fig. 6C.

Guillaume Lettre

(Remarks on code availability)

Referee #2

(Remarks to the Author)
In this study, the authors characterise mitochondrial DNA copy number/mutation across the All of Us and UKBB cohorts (around 620,000 individuals). Therefore, this is the largest characterisation of mtDNA copy number, SNVs and indels from bulk whole genome sequencing of blood and is a unique dataset. They first perform a nuclear GWAS to characterise associations with mtDNA copy number and variants. They then assess how these mutations relate to the presence of clonal haematopoiesis, concluding that age associated mtDNA SNVs, particularly those at low levels of heteroplasmy, are passenger events that only reach detection limits due to them hitch-hiking within clonal haematopoiesis expansions in blood.

The first sections of the results describe the nuclear GWAS on blood composition-corrected mtDNA copy number/ variants. These results are highly similar to what the authors published in 2023 albeit in a significantly expanded cohort. They validate their previous results, with some new additional associations. Overall, this part of the paper is not novel but is confirmatory.

Next, the authors describe, similar to what was shown in their previous paper, that there is an increased rate of SNV heteroplasmy with age. Of note, the numbers here are still very low – 0-1 heteroplasmic SNVs in young individuals, rising to 1-2 events in older individuals. There is a striking and convincing A>G and C>T bias on the heavy strand that is present for both synonymous and missense mutations suggesting these are not driven by positive selection. The authors suggest this is replication associated. It would be nice to see further validation of this hypothesis. The authors do show that there is increased mtDNA SNVs in those with haematological malignancies (Extended Data Fig 12) which is supportive. Are there additional sources of evidence? For example, does increased mtDNA SNV correlate with increased C>T at CpG, or shortened telomeres? Or perhaps some other lines of supporting data?

There is interesting dN/dS analysis suggesting that there is substantial negative selection of coding mtDNA mutations at high heteroplasmy as has been shown before. It would be good to understand the biological consequences of this negative selection further? Could this influence changes in mitochondrial clonal diversity with ageing or contribute to copy number differences over age?

The authors next identify that germline variants associated with CH also associate with 'high' mtDNA SNV burden. Personally, I found it difficult to follow to what extent associations with higher mtDNA SNVs were independent of CH and I struggled with the ordering of some of these analyses as I mention in a comment below.

By definition, bulk sequencing will only identify mutations that are either germline, or present in a clonal expansion and thus represented in sufficient cells to generate a sizeable fraction of mutant reads. Therefore, I find the idea that the majority of these mtDNA mutations will be passenger events within CH clones not surprising at all - the two-step model proposed thus seems to me like the baseline assumption, yet much emphasis is given to it in the abstract, introduction and discussion. What might be more interesting to ask is to what extent there may be selection acting on mutations in the mtDNA itself that are independent of clonal haematopoiesis and structuring the paper in this manner.

Specific comments:
-Can the authors comment on why the LofF mutations are predominantly C>T with fewer A>G? This contrasts with the data presented for synonymous and missense mutations (Fig 2E top panel and Extended Data Figure 6)

-Is it surprising that there is not much positive selection observed for mtDNA variants at lower levels of heteroplasmy? One can imagine that some somatic mtDNA mutations would confer a selective advantage to the cell, enabling it to proliferate faster. Or indeed, within the cell itself, some mutations may confer a selective advantage over other mitochondrial genomes within the same cell and thus their levels (and thus level of heteroplasmy) rise. Indeed, for negative selection to act on higher levels of heteroplasmy suggests that there must be positive selection acting on lower levels of heteroplasmy (or drift) to drive up those levels in the first place. Can the authors comment on whether they are able to interrogate for within-cell positive selection or across-cell positive selection at the level of mtDNA SNVs? Do the sites that are under negative selection at high heteroplasmy also display positive selection at low heteroplasmy?

- A caveat of this study is that it is unknown if low heteroplasmy mtDNA SNVs are present at higher heteroplasmy in few cells, or lower heteroplasmy across larger numbers of cells, as only the total number of mutant reads across all mtDNA reads in the bulk sample can be read out. Can the authors comment on how this might impact the interpretations in this study?

-Can the authors comment on the expected number of mtDNA mutations per mitochondrial genome, or per cell in blood from this study? For example, in those in whom there are clonal expansions, it becomes possible to detect all passenger mtDNA mutations within that clonal lineage. If individuals across different ages are identified with large clones, could this then provide a framework for estimating the per cell mtDNA mutation accumulation rate over time? Could this be modelled? It seems like a powerful dataset for doing so.

-Given the absolute levels as suggested by Fig 1 for mtDNA are very low even in the 'high' SNV burden (<2 SNVs per individual), can the authors comment on what sort of age-corrected mtDNA SNV level would be deemed high for it to be used as a sensitive marker of occult CH/haem malignancy as suggested by the authors?

-Given 50% of CH is 'driverless', and mtDNA SNV burden appears to be replication associated, can the authors identify driverless CH using high age-corrected mtDNA SNVs?

- Extended Data Figure 2 A/B – mtDNA CN – can the authors comment on the differences in CN between AoU and UKBB cohort? Why does there appear to be greater CN in younger females in UKBB? (Is the same seen in AoU? I could not find these data). Lastly, why does CN reduce with age?

-Extended Data Fig 12 showing increased mtDNA SNVs (age corrected) in haem cancers is interesting – should this be a main figure, perhaps combining the significant associations into an additional panel in Figure 4?

- Ordering of results. The section 'Germline variants that predispose to clonal hematopoiesis also predispose to high mtDNA heteroplasmy accrual' – is it worth simply looking at the correlation between CH and mtDNA SNV increase first, prior to a GWAS on increased mtDNA SNV burden? I note this section comes later 'High mtDNA SNV heteroplasmy co-occurs in samples with CH driver mutations', which shows a correlation between rare variants of CH and mtDNA SNV. To me, the first thing to do is to simply find all cases of CH (driver, driverless, copy number mosaic etc) and simply correlate with mtDNA SNV, prior to looking deeper at a GWAS or association with specific variants of CH. I felt this analysis was missing and critical given the conclusion that higher mtDNA SNV reflects hitch-hiking with CH.

(Remarks on code availability)
I have not had a chance to review the code for this study.

Referee #3

(Remarks to the Author)
This study leverages the All of Us and UK Biobank datasets to investigate the origins of age-related mtDNA mutations in blood. This work employs numerous approaches to correct the data, enabling this extensive study. The research shows that clonal hematopoiesis (CH) creates an isolating or magnifying effect, revealing low-level mutations. Characterization of these variants suggests that the primary cause of mtDNA mutation with age is deamination in the displaced/heavy strand, with polymerase error being secondary. These mutations do not appear to be under any selection, indicating that they do not affect mitochondrial function. This finding is significant, as I ultimately agree that mtDNA mutations are not a driver of normal aging. Even in a clonal expansion condition with nuclear driver variants, these mutations do not reach a high enough burden. Thus, mtDNA mutation accumulation is an aging hallmark. This conclusion would be strengthened if individual cells carrying driver mutations were isolated and sequenced at a single-cell level, but that is merely a nice-to-have and not necessary to support the findings.
Some Points of Novelty:
Several aspects of this study are novel
-Updated mtSwirl (v2) algorithm.
-Largest databases to date
-corrects for mtDNA copy number and distribution of cell types
-corrects for ancestry
-Regresses heteroplasmy to driver nuclear variations and CH. To an extent, this action had to find CH at the core, but that doesn't make it irrelevant or error-prone.
Concerns:
Some issues arise from the manuscript. It was unclear how novel the elements of this study were in the context of the existing literature. Concerns arose when it was observed that the types of age-related mutations had been previously described, and the nuclear mutations driving CH were largely understood. However, I cannot find where the aging mtDNA variants can be highlighted by CH. Driver genes have been well described in CH, but they have not been leveraged to highlight mtDNA mutations. I believe that the aggregate of these finding bear support, especially in light of the number of tools leveraged to correct the data. This work should provide the definitive description of age-related variation and may inspire new studies on mtDNA variation, CH, and aging in disease conditions.
Isolated points:
Additional contributions are two new driver genes in CH. It is this reviewer's opinion that this might be more strongly highlighted in the text.
The connection with clonal hematopoiesis and telomere-associated genes is interesting. Given that changes in telomere genes alter clonal hematopoiesis indirectly via telomere length. It would be interesting to test the association with telomere

length and the accumulation of age-associated mitochondria variants.
While it is likely out of the scope of the current manuscript, it would be an interesting comparison to examine the mitochondrial variants in two tissues, one hematopoietic and one non-hematopoietic, from the same individual to determine if the same variants are present. This experiment would inform when the variants likely arose.
The literature is in dire need of a reset on the role of oxidative stress-driven mutations in aging. The authors are invited to strengthen this issue in the manuscript and reduce other redundancies in the discussion.
CH could significantly influence the variable presentation of phenotypes in mtDNA disease patients. This concept may deserve a substantial comment from the authors in the discussion.
Minor comments for the authors:
- This manuscript is generally well written. I suggest reducing redundancy between results and discussion and incorporating some emphatic language to emphasize the novelty and significance of the findings. The manuscript feels understated.
-Sentence construction using this or these as the lone subject is prone to confusion. Please augment these sentences with a noun for better connectivity. Examples include line 187, but this is not comprehensive
-I believe the word “strand” is missing from line 199.
-In general, the manuscript is written to a mitochondrially-focused audience and may not be accessible to the broad readership of Nature. For example, most readers may not be familiar with references to the heavy strand in the mitochondrial genome or OL. Some context for these concepts would improve the readability.

-The authors should be careful with terminology around “mutations” and “variants”. In general, “variant” is preferred. If both are used, “mutation” should refer to changes that have known negative effects. For example, the authors use the term “TERT mutation” to refer to variants (SNPs) that are found in and around the TERT gene and are associated with specific phenotypes. For the majority of these variants, no functional studies have been performed to understand their role and it is unclear if they are functional or in disequilibrium with the functional variant.

(Remarks on code availability)
I was unable to review the code. This should be done via specialist review.

Referee #4

(Remarks to the Author)
I co-reviewed this manuscript with one of the reviewers who provided the listed reports.

(Remarks on code availability)

Version 2:

Reviewer comments:

Referee #1

(Remarks to the Author)
The authors have addressed my comments appropriately.
Guillaume Lettre

(Remarks on code availability)

Referee #2

(Remarks to the Author)
The concept that passenger mitochondrial mutations are detectable in blood due to them hitchhiking with clonal haematopoiesis seems expected but has not been confirmed at population level before. This manuscript carefully shows that this is indeed the case and that such mitochondrial mutations accrue due to replication errors. The authors show that there is no evidence of mitochondrial 'hypermutation' driven by germline nuclear variants, and that there is an absence of specific mitochondrial mutations driving positive selection in blood over age. These results are important and clarify confusion in the field around any causal contribution of mitochondrial mutations to mutational landscapes and clonal architecture in blood. The dataset is the largest to date, with validation using independent datasets, and will be relevant to several research communities.

Thank you for addressing my queries. I have no further comments.

(Remarks on code availability)

Referee #3

(Remarks to the Author)
The authors were responsive to the original positive review.

I have minor points for the authors:
1) mtCN is never defined in the manuscript.
2) The text “Given the rich literature linking telomerase and aging…” would be more accurately stated “Given the rich literature linking telomere length and aging…”. Telomere length shortens with age.
3) Regarding mutation and variant, there may be a tendency to see "mutation" as impactful and "variant" as unimpactful. I would suggest that age-acquired mutations be initially described as neutral mutations to prevent this erroneous connection for general readers.

(Remarks on code availability)

Referee #4

(Remarks to the Author)
I co-reviewed this manuscript with one of the reviewers who provided the listed reports.

(Remarks on code availability)

Open Access This Peer Review File is licensed under a Creative Commons Attribution 4.0 International License, which permits use, sharing, adaptation, distribution and reproduction in any medium or format, as long as you give appropriate credit to the original author(s) and the source, provide a link to the Creative Commons license, and indicate if changes were made.
In cases where reviewers are anonymous, credit should be given to 'Anonymous Referee' and the source.
The images or other third party material in this Peer Review File are included in the article’s Creative Commons license, unless indicated otherwise in a credit line to the material. If material is not included in the article’s Creative Commons license and your intended use is not permitted by statutory regulation or exceeds the permitted use, you will need to obtain permission directly from the copyright holder.
To view a copy of this license, visit https://creativecommons.org/licenses/by/4.0/

Referee expertise:

Referee #1: human genetics

Referee #2: somatic evolution

Referee #3: mitochondrial DNA

Referees' comments:

Referee #1 (Remarks to the Author):

In this manuscript, Gupta and colleagues build on their previous work and address a long-standing question in the field of mitochondrial biology: Why does the mitochondrial genome accumulates mutations as we age? To do so, the authors analyze WGS data from >700K participants from the AoU and UKBB cohorts. In the first part of the manuscript, they assessed the quality of their new mitochondrial genomic dataset by extending many analyses performed in their earlier study. Using GWAS, they replicated and identified many loci associated with mitochondrial copy number and mtDNA variant heteroplasmy, highlighting many strong candidate genes. Subsequent analyses are more interesting and novel. They suggested that age-accumulating variants are unlikely due to oxidative stress, probably somatic in nature, and not under positive selection. One of the most insightful observations came from a GWAS of mtDNA SNV burden, which identified several loci also associated with clonal hematopoiesis (CH). This and other observations led the authors to propose a model by which heteroplasmy increases during aging as a consequence of the proliferative advantage confers by CH driver somatic mutations.

The strengths of the study include the very large sample size, the consistency of the results between AoU and UKBB, a strong analytical plan and a well-written manuscript. While the first part of the paper is an extension of the previous work, it serves to validate the call set. The molecular model (CH leading to increase heteroplasmy) is plausible and well-supported by the data. However, it does raise the question of the clinical (causal) relevance of accumulating heteroplasmic variants in aging and diseases.

Thank you for your positive feedback.

I have only a few comments:

1. Do the main results indicate that age-related accumulation of mtDNA SNV is not particularly important for human health, but rather a bystander product of CH? Can the authors extend on this in the Discussion? What are the implications of these results for mitochondrial diseases? How should researchers working in this field consider mtDNA SNV? Should they be ignored? Should the focus be only

on mtDNA indels? The study is important and will impact the field, so guidance should be provided.

There is little doubt that high levels of certain mtDNA variants, acquired through maternal transmission or somatically, can contribute causally to severe disease pathology. Most disease-causing mtDNA variants (which can be SNVs, indels, or large re-arrangements) tend to be high in heteroplasmy and tend to disrupt protein or RNA function. On the other end of the spectrum, age associated accrual of mutations in mtDNA has also been documented in many tissues for decades, and this process has been proposed widely to contribute causally to common age-associated disease. Our current study, which is focused on rapidly proliferating blood cells, confirms that accrual of heteroplasmic mtSNVs is an excellent biomarker of aging, however, we find little or no association with common age associated diseases. The mtSNV heteroplasmies that accumulate with age, in contrast to known pathogenic disease-causing mtDNA variants, are dominated by neutral variants which act as passenger mutations and increase in abundance due to nucDNA driver mutations.

Further, many studies of rare pathogenic mtDNA heteroplasmy have focused on solid terminally differentiated tissues such as brain, heart, and skeletal muscle. As we have focused on blood, future studies will be required to rigorously determine the cause of mtDNA mutation accrual in solid organs, and whether they also simply represent biomarkers of aging or whether they contribute causally to disease in those tissues.

We have now elaborated on the above in the Discussion.

2. What is known about the impact of mtDNA mutations on mitochondrial functions? I guess that somatic heteroplasmy variants have little/no impact (based on the selection analyses). What about the maternally-inherited variants?

Heteroplasmic mtDNA mutations can be maternally inherited or somatically acquired. Regardless of the origin of the mutation, the consequence of the mutation (*e.g.*, silent versus pathogenic) is dependent on (i) the variant itself, (ii) its heteroplasmy level, and (iii) the environmental / metabolic context. In general, low heteroplasmy variants are far less likely to have any impact as mtDNA variants tend to be functionally recessive (*i.e.*, a sufficiently high heteroplasmy is required for a variant to lead to a functional consequence). In contrast, high heteroplasmy missense and nonsense mtDNA variation can contribute causally to severe disease pathology. We believe that unlike known pathogenic mutations (which tend to be rare and under negative selection), the bulk of the variants identified in our study tend to be neutral. Thus, the observed mtSNV somatic variant burden metric is dominated by neutral variants. This does not preclude the presence of consequential pathogenic mtDNA mutations (which may arise somatically or may be inherited). We have updated the discussion (see response to #1) to reflect and clarify this.

3. The suggestion that mtDNA SNV burden could be earlier biomarkers of CH is interesting, and could be tested. For instance, after removing

known CH carriers, is the mtDNA SNV burden still associated with hematologic cancers?

Thank you for this excellent suggestion. The association persists when we remove the 17,504 individuals with documented CH. We have updated our text to include:

"Two lines of evidence demonstrate that mtSNV burden may represent an earlier and more sensitive biomarker of CH. First, GWAS and RVAS for mtSNV burden point to nuclear germline variants that predispose to CH, even after removing the 17,504 individuals with documented CH (Figure 3A, 3B). Second, when we remove these individuals within UKB (N = 11,465), the association between mtSNV burden and hematologic cancer also persists with an odds ratio of 1.27 ($P = 3.06 * 10^{-47}$) versus 1.29 ($P = 2.33 * 10^{-59}$) with all samples."

4. Potentially using the UKBB proteomic data and excluding the known CH carriers, do participants with a high mtDNA SNV burden have higher levels of pro-inflammatory cytokines (e.g. IL6, TNFa), as observed in CH carriers (after correcting for age)?

Thank you for these great suggestions.

We have now obtained the values of the pro-inflammatory cytokines (IL1a, IL6, TNFa), aging biomarkers (FGF21, GDF15), and telomere length (per Reviewers 2 and 3). We performed correlations between these markers and mtSNV count. We attempted the analsysi with and without CH carriers and found that the results were similar. We also looked at associations with $mtCN_{adj}$. We have now updated our text with a new section and a new extended data figure detailing our findings:

"Given the rich literature linking telomerase and aging, we next evaluated for evidence of colocalization of GWAS signal in the *TERT* locus for mtCN (Extended Data Figure 2C) and mtDNA SNV burden (Figure 3A). The variant rs7705526 (chr5:1285859:C:A) had a posterior inclusion probability (via fine-mapping) of ~1 for mtDNA SNV burden and 0.39 for mtCN suggesting co-localization (colocalization posterior probability, CLPP = ~0.39). This association also colocalizes with a recent GWAS of clonal hematopoiesis[28] (CLPP = 1) and a recent GWAS of telomere length[31] (CLPP = 1). Thus, based on our current work and prior studies, this *TERT* variant: (i) increases $mtCN_{adj}$, (ii) increases mtDNA SNV burden, (iii) increases CH risk, and (iv) increases telomere length.

We obtained telomere lengths from UKB and explored their correlation with mtDNA phenotypes (Methods). After correction for covariates including age, we observed a strong positive correlation between $mtCN_{adj}$ and telomere length (Extended Data Figure 15A). In contrast, mtSNV burden did not correlate with telomere length. This is not entirely unexpected – recent work has shown that genetic predisposition to longer telomere length actually associates with higher CH, while CH subsequently results in a decline in telomere length[32]. In agreement, our fine mapping indicates that the likely causal variant at *TERT* increases telomere length and CH risk. This same variant increases mtSNV count. We speculate that a genetic predisposition for longer telomeres may increase mtSNV count by increasing CH predisposition, however this effect is masked by the fact that CH itself results

in more cell replications which reduces telomere length but increases heteroplasmic mtSNV count.

We also correlated mtDNA phenotypes with circulating levels of age-associated markers (GDF15, FGF21) and inflammatory cytokines (IL1A, IL6, TNF). Even after correction for age, we observe a significant inverse relationship between GDF15 and $mtCN_{adj}$ (Extended Data Figure 15A), which may indicate a shared underlying mechanism of variation; indeed GDF15 was identified as a correlate of an inborn mitochondrial disease[33]. We also observe that higher $mtCN_{adj}$ correlates with lower IL6 and TNF, likely due to residual effects of inflammatory pathways in blood $mtCN_{adj}$ despite compositional correction. These protein biomarkers do not show significant correlation with mtSNV burden, likely indicating an independent process (i.e., CH due to nucDNA variation).”

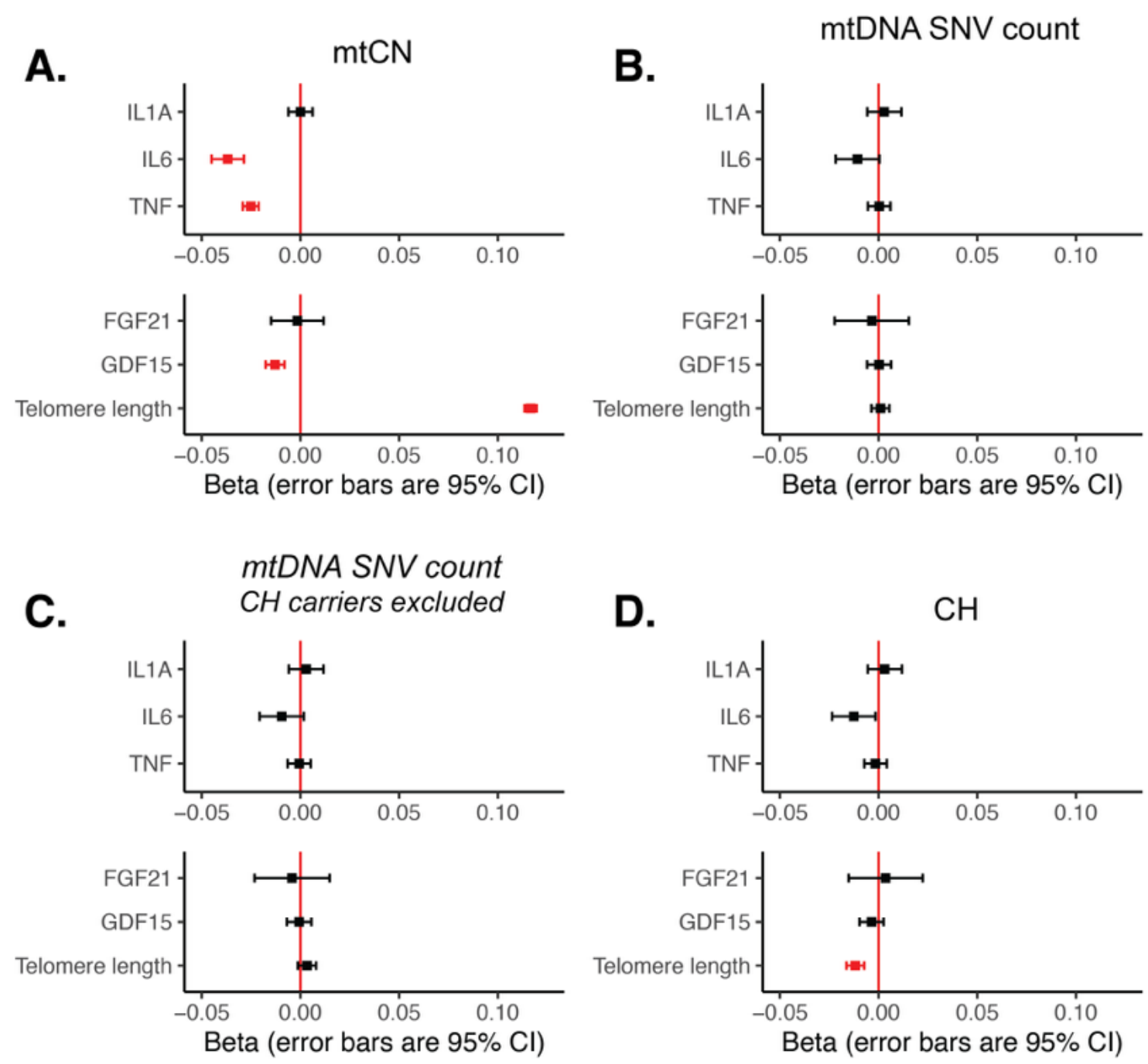

Extended Data Figure 15. Correlations between mtDNA traits and CH and biomarkers of aging and inflammation. Regression betas of inflammatory biomarkers (upper panel: IL1A, IL6, TNF as measured by proteomics) and aging / reductive stress biomarkers (FGF21 and GDF15 via proteomics and telomere length via PCR) onto **A.** $mtCN_{adj}$, **B.** mtSNV count, **C.** mtSNV count with CH carriers excluded, and **D.** CH. In all analyses, corrections were performed by including age, age^2, sex, age*sex, age^2*sex, genetic ancestry, and haplogroup in the association model. Red line is beta = 0. Red points indicate associations that pass Bonferroni multiple testing correction (N = 6 tests per analysis). Error bars correspond to 95% CI.

5. Mediation analyses. Does the accumulation of mtDNA SNV mediate the effect of CH on blood cancer risk? I would think the answer is no, but it would be nice to show.

Several lines of evidence in this study point to the notion that mtSNV burden may be identifying individuals with latent CH who are not identified as having CH. See our response to your comment #3 – for instance, GWAS and RVAS for mtSNV burden point to nuclear germline variants that predispose to CH even after removing the 17,504 individuals

with documented CH. Further, we see a correlation between hematologic cancer and mtSNV burden despite removal of individuals with detect CH. Thus, we suspect that there is latent CH that is not measured fully by CH detection using nuclear WGS, and mtSNV burden is likely capturing a portion of this latent variable that the CH measurement itself is not. This implies that detection of "mediation" of mtSNV burden between CH and hematologic cancers would occur even if no such mediation existed. Indeed, the regression of mtSNV burden on CH has a coefficient of 0.187 with a p-value < 2e-16. The regression of heme cancer onto mtSNV burden and CH produces coefficients of 0.4421 and 0.6313 respectively, with p-values of < 2e-16 and 1.05e-12 respectively, implying "mediation".

We expect CH to correlate with elevated hematologic cancer risk. Given that both the measured CH and mtSNV burden are likely measuring latent CH, CH and mtSNV burden will be correlated. Supporting this notion, as described in the response to your point 6, we find genetic evidence suggesting that CH impacts mtSNV burden, and not vice versa. Thus, mtSNV burden will correlate with hematologic cancer risk as well. Since mtSNV burden is likely capturing different samples with latent CH than the measurement of CH itself, mediation will be present and not complete even though no true mediation is occurring, exactly as observed.

We also used bivariate linkage disequilibrium score regression (LDSC) to estimate the genome-wide genetic correlation between mtSNV burden and CH. The estimated genetic correlation was 1.0473 with an estimated standard error of 0.2778. This suggests almost perfect sharing of genetic architecture between mtSNV burden and CH even across genetic loci that are not genome-wide significant, strengthening the argument that they are likely measuring the same underlying latent variable. However, the heritability estimate of mtSNV burden had a Z-score of ~1, which has been shown to lead to noisy genetic correlation estimates. Thus we did not include this analysis in the main text.

6. Along the same line, I think that Fig.3B captures the MR analysis performed by the authors, but it is not explicitly described in the text (there is one paragraph in the Methods). Note that bi-directional MR would be informative to re-enforce the model that CH causes age-accumulating mtDNA SNV (and not the other way around).

We have now explicitly described our analyses as Mendelian Randomization in the text and have included the reverse regression (completing the bi-directional analysis) as well with the associated plots in the Extended Data:

"We next performed Mendelian Randomization between mtSNV burden and CH. Across virtually all variants associated with CH at GWS[28], we observed a positive correlation with corresponding effect sizes for mtDNA SNV burden (Figure 3C, Methods), indicating that CH increases mtDNA SNV burden. The converse was not true (Extended Data Figure 10), suggesting that mtDNA SNV burden does not impact CH risk."

7. The authors analyzed genetically diverse cohorts but did not detailed how many participants from each genetically-defined groups were considered (or I missed it). Were they ancestry-specific significant GWAS findings (which would not simply be due to power)?

We have now updated the supplemental tables to include a breakdown for each trait of the per-population samples size for heteroplasmy traits (see Supplementary Tables 1 and 2). While diverse populations analysis is a core part of our analytical pipeline and greatly adds to our power, we believe that a detailed analysis of ancestry-specific GWAS is outside the scope of this study.

8. (Minor). Fig. 2C. Add scale on x-axis. Same for Ext. Fig. 6C.

We have made these corrections.

Guillaume Lettre

Referee #2 (Remarks to the Author):

In this study, the authors characterise mitochondrial DNA copy number/mutation across the All of Us and UKBB cohorts (around 620,000 individuals). Therefore, this is the largest characterisation of mtDNA copy number, SNVs and indels from bulk whole genome sequencing of blood and is a unique dataset. They first perform a nuclear GWAS to characterise associations with mtDNA copy number and variants. They then assess how these mutations relate to the presence of clonal haematopoiesis, concluding that age associated mtDNA SNVs, particularly those at low levels of heteroplasmy, are passenger events that only reach detection limits due to them hitch-hiking within clonal haematopoiesis expansions in blood.

The first sections of the results describe the nuclear GWAS on blood composition-corrected mtDNA copy number/ variants. These results are highly similar to what the authors published in 2023 albeit in a significantly expanded cohort. They validate their previous results, with some new additional associations. Overall, this part of the paper is not novel but is confirmatory.

Next, the authors describe, similar to what was shown in their previous paper, that there is an increased rate of SNV heteroplasmy with age. Of note, the numbers here are still very low – 0-1 heteroplasmic SNVs in young individuals, rising to 1-2 events in older individuals. There is a striking and convincing A>G and C>T bias on the heavy strand that is present for both synonymous and missense mutations suggesting these are not driven by positive selection. The authors suggest this is replication associated.

It would be nice to see further validation of this hypothesis.

The mtDNA replicates via a strand displacement model, and because of this asymmetry, gives rise to a strand-specific mutational spectrum A>G and C>T likely due to polymerase errors or deamination. This mutational spectrum has been widely described in the past and is what we observe as accruing with age in blood across two biobanks. We have cited the relevant literature including Kennedy et al. 2013, Ju et al. 2014.

The authors do show that there is increased mtDNA SNVs in those with haematological malignancies (Extended Data Fig 12) which is supportive. Are there additional sources of evidence? For example, does increased mtDNA SNV correlate with increased C>T at CpG, or shortened telomeres? Or perhaps some other lines of supporting data?

We provide multiple lines of evidence in support of our mechanistic model (which reviewers 1 and 3 state is well supported):

1. **As stated above, the mutational spectrum and strand bias of the age accruing variants is exactly what is classically described for mtDNA replication. The spectrum does not align with overt ROS damage (which would be expected to cause predominantly C>A transversions). The spectrum also does not appear consistent with transcription-coupled error as we describe in the manuscript.**
2. **To discover the underlying mechanism, we treated mtSNV as a "trait" and performed GWAS. We predominantly recover nuclear loci associated with CH. We use Mendelian Randomization to show that CH causes the observed increase in mtSNV count, rather than vice versa. RVAS for the same trait identifies numerous pLoF variants in CH driver genes as associated; many of these are likely nucDNA somatic driver mutations for CH. In other words, we can use mtSNV count to discover CH driver mutations.**
3. **In terms of disease, mtSNV count is associated primarily with hematologic cancer risk. After identifying individuals with CH using WGS, we find that people with CH tend to have substantially higher mtSNV burden at any given age.**
4. **All of the above persists despite removal of individuals annotated as having CH: the GWAS and RVAS association landscapes still show predominantly CH-related variants as associated with mtSNV count, and mtSNV count is still primarily associated with hematologic malignancy. This implies that mtSNV count is identifying individuals with CH who are not identified using typical WGS-based methods.**

Thank you for suggesting that we dig deeper into the relationship with telomere length – Reviewer #3 also asked that we do so. We have now updated our revised manuscript with these two paragraphs:

"Given the rich literature linking telomerase and aging, we next evaluated for evidence of colocalization of GWAS signal in the *TERT* locus for mtCN (Extended Data Figure 2C) and mtDNA SNV burden (Figure 3A). The variant rs7705526 (chr5:1285859:C:A) had a posterior inclusion probability (via fine-mapping) of ~1 for mtDNA SNV burden and 0.39 for mtCN suggesting co-localization (colocalization posterior probability, CLPP = ~0.39). This association also colocalizes with a recent GWAS of clonal hematopoiesis[28] (CLPP = 1) and a recent GWAS of telomere length[31] (CLPP = 1). Thus, based on our current work and prior studies, this *TERT* variant: (i) increases $mtCN_{adj}$, (ii) increases mtDNA SNV burden, (iii) increases CH risk, and (iv) increases telomere length.

We obtained telomere lengths from UKB and explored their correlation with mtDNA phenotypes (Methods). After correction for covariates including age, we observed a strong positive correlation between $mtCN_{adj}$ and telomere length (Extended Data Figure 15A). In contrast, mtSNV burden did not correlate with telomere length. This is not entirely unexpected – recent work has shown that genetic predisposition to longer telomere length actually associates with higher CH, while CH subsequently results in a decline in telomere length[32]. In agreement, our fine mapping indicates that the likely causal variant at *TERT* increases telomere length and CH risk. This same variant increases mtSNV count. We speculate that a genetic predisposition for longer telomeres may increase mtSNV count by increasing CH predisposition, however this effect is masked by the fact that CH itself results in more cell replications which reduces telomere length but increases heteroplasmic mtSNV count."

There is interesting dN/dS analysis suggesting that there is substantial negative selection of coding mtDNA mutations at high heteroplasmy as has been shown before. It would be good to understand the biological consequences of this negative selection further? Could this influence changes in mitochondrial clonal diversity with ageing or contribute to copy number differences over age?

These are all interesting possibilities but will require population-scale, single-cell mtDNA heteroplasmy analysis and are beyond the scope of the current paper.

The authors next identify that germline variants associated with CH also associate with 'high' mtDNA SNV burden. Personally, I found it difficult to follow to what extent associations with higher mtDNA SNVs were independent of CH and I struggled with the ordering of some of these analyses as I mention in a comment below.

Having worked for decades in the field of mitochondrial genetics, going into this analysis, we were expecting our GWAS/RVAS to pick up variants in nuclear genes encoding mtDNA replication machinery (resulting in a subtle "polymerase mutator" phenotype). However, after using mtSNV burden as a "trait" and performing GWAS, we are reporting that the heritable component of this trait in humans is driven in large part by nuclear loci that are not related to mitochondria but rather related to CH risk. This was a wholly unexpected / unanticipated result for the mitochondrial community. Further, across all tested domains of human disease, mtSNV count is primarily associated with blood cancers, as seen with CH. Our data point to the observed age-accumulation of mtSNV count in blood arising largely from passenger effects from CH. Even when we exclude the 17,504 individuals identified as having CH across both biobanks, we find that the genetic loci associated with mtSNV count (as well as association with hematologic malignancy) remain the same, implying that mtSNV count elevation can identify CH even when CH is not detected using traditional methods. Our work suggests that mtSNV may be a more sensitive marker of CH, as pointed out by Reviewer #1. Please note that this is a very original result for the mitochondrial field.

By definition, bulk sequencing will only identify mutations that are either germline, or present in a clonal expansion and thus represented in sufficient cells to generate a sizeable fraction of mutant reads. Therefore, I find the idea that the majority of these mtDNA mutations will be passenger events within CH clones not surprising at all - the two-step model proposed thus seems to me like the baseline assumption, yet much emphasis is given to it in the abstract, introduction and discussion.

You state that this was your "baseline assumption" – but that is not the case for the mitochondrial research community nor for the aging community – and you will not find any papers reporting our results.

We agree that once we report our mechanism, it seems both plausible and obvious, as our result is so robust. However, prior to our work, the mechanistic basis for this accrual was not known, and CH as a cause of the rise in mtSNV burden with age had not been discussed to our knowledge. Reviewer #3 appropriately states "The literature is in dire need of a reset on the role of oxidative stress-driven mutations in aging." Reviewers 1 and 3 point out our study is novel and have recommended that we expand the discussion of our conclusions. As stated by Nils-Goran Larsson, a respected scientist in the field, in 2013 review: "…it remains unclear how mtDNA mutations accumulate and how they are relevant to aging, as mtDNA mutations are present at relatively low overall levels in normal aging tissues."

A bit of background might be instructive. The accrual of mtDNA mutations with age has been noted for decades, and it has passively been assumed that the accumulation of these mutations is causal for age-associated disease. However, there has never been a clear mechanistic explanation for the accumulation of these mutations nor strong proof of their disease relevance. It was "believed" that reactive oxygen species (ROS) produced as part of the mitochondrial electron transport chain (ETC) produced mitochondrial DNA mutations, which then resulted in a vicious cycle of dysfunctional ETC complexes producing more ROS and so forth (see Harman 1972, Wallace 1999). For decades the field has focused on this free radical theory, but starting ten years ago, studies in brain (Kennedy et al. 2013) using sensitive sequencing methods began to cast doubt on this model as most of the accumulating lesions in the brain were not oxidative in nature, and rather, were more consistent with replication errors.

This, however, did not explain the mechanism of accrual with age, nor did it explain how these mutations become abundant enough to be detected as a function of age using basic WGS. Given we found that much of the nuclear genetic architecture controlling mtDNA copy number and inherited mtDNA length heteroplasmies implicated mtDNA replication machinery, our prior expectation was that GWAS of mtSNV count would point to nuclear loci involved in mtDNA replication (e.g., mitochondrial polymerase *POLG*, etc.) and the generation of mtDNA mutations.

Instead, our analyses suggest that the nuclear variants contributing the largest component to the heritability of mtSNV burden act via CH. Moreover, our work confirms that the

accumulation of mtSNV burden is an excellent aging biomarker and clarifies the mechanism as to why it is observed to increase with age (namely, it is driven by CH which is an age-dependent process). We have little evidence that it is associated with common diseases beyond the risk of hematologic malignancies.

Based on this, we believe our structuring of our manuscript is logical and intuitive for both the broad readership of *Nature* as well as the specialists in mitochondrial DNA biology, somatic genetics, and aging.

What might be more interesting to ask is to what extent there may be selection acting on mutations in the mtDNA itself that are independent of clonal haematopoiesis and structuring the paper in this manner.

We agree that the question of how selection for/against mtDNA variants could modify and/or act independently of CH is interesting. In our data, we believe that many of the mtDNA variants identified are acting neutrally. However it is certainly possible for mtDNA variants to exert selective forces – we previously reported in *NEJM* that in maternally transmitted mtDNA diseases, there is evidence of lineage-specific purification against pathogenic mtDNA heteroplasmy (Walker et al. 2020 *NEJM*). We believe that in the future it will be fascinating to perform population-scale single cell mtDNA heteroplasmy analysis to pursue the types of studies you propose. We now include the following sentence in the paper:

"We anticipate that population-scale, single-cell analysis will likely be required to further extend our work, identify if low heteroplasmy variants in bulk are low heteroplasmy in single cells, and uncover interactions between selection acting on somatic mtDNA variants and the impact of CH driver mutations."

Specific comments:
-Can the authors comment on why the LofF mutations are predominantly C>T with fewer A>G? This contrasts with the data presented for synonymous and missense mutations (Fig 2E top panel and Extended Data Figure 6)

For the purpose of this analysis, "loss of function" mtSNVs are defined as those causing a stop-gained mutation (i.e., a nonsense mutation). We can consider the heavy strand first – as you have noted, in UKB and AoU, heavy strand C>T and A>G mutations are much more common, consistent with the strand displacement model. We generated a list of all possible mtDNA mutations (49,704 variants) and then used VEP to determine the consequence of each of these mutations. Using all possible mtDNA SNVs, we find that there are 179 possible C>T heavy strand mutations that lead to predicted LoF effect. In contrast, there are only 2 A>G heavy strand mutations that lead to LoF. Even though we scale the observed rate of mutations of each class by the total number of possible mutations of that class, we are simply unlikely to observe mutations in the A>G heavy LoF class given that only 2 mutations can lead to this – indeed at our sample size the observed rate is 0.

The reason for why there are many more possible C>T LoF mutations than A>G is because (1) most proteins are encoded on the mtDNA heavy strand and (2) in mtDNA, stop codons are AGA and AGG. In this analysis, loss of function SNVs are defined as nonsense mutations. As most proteins in mtDNA are encoded on the heavy strand, the sequence of the mRNA for those proteins arises from the sequence of the light strand. A>G heavy strand mutations lead to U>C changes in the mRNA which cannot produce nonsense mutations. In contrast, C>T heavy mutations produce G>A changes which can lead to nonsense mutations (for instance, GGA>AGA is a Gly>Ter mutation). The 2 possible nonsense mutations arising from A>G heavy mutations are due to ND6 which is the only protein-coding gene encoded on the light strand.

Turning to the light strand, there are 116 possible C>T light nonsense mutations and 93 possible A>G light mutations. Corresponding to this, the observed mutation counts between the two classes are rather similar. In UKB we observe 5 heteroplasmic LoF A>G light mutations and 2 heteroplasmic LoF C>T light mutations.

-Is it surprising that there is not much positive selection observed for mtDNA variants at lower levels of heteroplasmy?

This is not very surprising. It has been shown repeatedly that functional consequences of mtDNA heteroplasmic variation depend on the heteroplasmy level of those variants. This is termed the "heteroplasmy threshold effect" (Shoffner et al. 1990 *Cell*, Rossignol et al. 2003 *Biochem J*) and has been seen in numerous inborn mitochondrial disorders. We have shown that after introducing a pathogenic variant in cell culture, purifying selection was only observed when heteroplasmy was sufficiently high (Kotrys et al. 2024 *Nature*). In other words, heteroplasmic pathogenic mtDNA variants tend to be functionally recessive. Thus, we believe that the norm is for drift to take mtDNA variants to a sufficiently high heteroplasmy before selective forces begin acting on these variants.

One can imagine that some somatic mtDNA mutations would confer a selective advantage to the cell, enabling it to proliferate faster.

This is indeed possible – there are certain tumors (oncocytomas) in which we have shown that mtDNA mutations in complex I genes at extremely high levels of heteroplasmy (homoplasmy) can fulfill criteria as drivers (Gopal et al. 2018 *PNAS*, Gopal et al. 2018 Cancer Cell). We emphasize that the driver mtDNA mutations in this work were found to all have high heteroplasmy. We interpret this as implying that oncocytoma driver mutations in mtDNA likely achieved a sufficiently high heteroplasmy prior to driving proliferation of tumor and undergoing positive selection.

Or indeed, within the cell itself, some mutations may confer a selective advantage over other mitochondrial genomes within the same cell and thus their levels (and thus level of heteroplasmy) rise. Indeed, for negative selection to act on higher levels of heteroplasmy suggests that there must be positive selection acting on lower levels of heteroplasmy (or drift) to drive up those levels in the first place. Can the authors comment on whether they are able to interrogate for within-cell positive selection or across-cell positive selection

at the level of mtDNA SNVs? Do the sites that are under negative selection at high heteroplasmy also display positive selection at low heteroplasmy?

In this study, we have focused on dynamics of accrual of heteroplasmy count, rather than heteroplasmy itself, as a function of age. The nuclear genetic effect on heteroplasmy count is swamped by CH. Future analyses will be required to detect more subtle effects that act independently of clonal processes. As you have suggested, single-cell analyses would likely be required to comment on within-cell positive selection for individual variants.

We believe there is strong evidence for drift as the cause for certain variants achieving high heteroplasmy. We find that missense variants are far more frequent at low heteroplasmy relative to synonymous variants, with their frequency falling with higher heteroplasmy. Our dN/dS results are aligned with this and do not suggest positive selection even at low heteroplasmy when stratified by mutation class or gene. As described above, the mitochondrial "heteroplasmy threshold effect" is an established phenomenon in which pathogenic variants can be present without consequence until they reach a sufficiently high fraction i.e. due to drift. Our results are in agreement with the action of this phenomenon.

We do not comment on specific heteroplasmic SNVs, however we note that of 16,747 unique SNVs outside the Ori region, only 26 are found in more than 300 people in our dataset and with >50% of the variants being found in 5 or fewer individuals across both biobanks. There are a very limited number of recurrently mutant sites. On closer review of the most frequently heteroplasmic variants, there is little evidence supporting the notion that any of these variants are present in most people with CH, though we do see that age-accumulating class variants tend to be present more frequently in CH as shown in Figure 5. We focus on collapsing analyses e.g., grouping variants by gene and variant class.

- A caveat of this study is that it is unknown if low heteroplasmy mtDNA SNVs are present at higher heteroplasmy in few cells, or lower heteroplasmy across larger numbers of cells, as only the total number of mutant reads across all mtDNA reads in the bulk sample can be read out. Can the authors comment on how this might impact the interpretations in this study?

Thank you for pointing this out – we agree that this is a limitation of our study and have included a brief discussion on this in our "Limitations" section:

"Third, because all analyses occur in bulk samples, we are unable to evaluate the heteroplasmy distribution of a particular variant across cells in each sample and cannot disentangle if individuals with multiple mtSNVs have a single CH clone carrying all SNVs or multiple clones carrying each mutation. Single-cell analysis would certainly provide additional resolution to tease this apart. However, we note that our core results showcasing the overall mutational spectrum, our observation that higher heteroplasmy variants are subject to higher levels of negative selection, and our nuclear genetic results would not be affected. We anticipate that population-scale, single-cell analysis will likely be required to further extend our work, identify if low heteroplasmy variants in bulk are low heteroplasmy in single cells, and uncover interactions between selection acting on somatic mtDNA variants and the impact of CH driver mutations."

-Can the authors comment on the expected number of mtDNA mutations per mitochondrial genome, or per cell in blood from this study? For example, in those in whom there are clonal expansions, it becomes possible to detect all passenger mtDNA mutations within that clonal lineage. If individuals across different ages are identified with large clones, could this then provide a framework for estimating the per cell mtDNA mutation accumulation rate over time? Could this be modelled? It seems like a powerful dataset for doing so.

We agree this could be an exciting idea to pursue however is beyond the scope of the current paper. To validate such modeling, it would be important to have large single cell datasets from individuals of varying ages in whom mtSNV count can also be quantified – the availability of such data is limited at this time and to generate such a dataset is not within scope of this work.

-Given the absolute levels as suggested by Fig 1 for mtDNA are very low even in the 'high' SNV burden (<2 SNVs per individual), can the authors comment on what sort of age-corrected mtDNA SNV level would be deemed high for it to be used as a sensitive marker of occult CH/haem malignancy as suggested by the authors?

In our data in both UKB and AoU, using an mtSNV count of 1 or more as "high" maximizes the Youden index for the identification of individuals with CH. However, the accuracy of the resultant sensitivity and specificity indices is limited. First, as you have implied, these metrics depend on the distribution of mtSNV count in a given dataset, which is primarily a function of mtCN and sequencing depth. AoU has a higher average mtCN and indeed his cutoff yields a higher sensitivity for detection of CH in AoU versus in UKB Second, our claim that mtSNV burden may be a more sensitive measure of CH than current approaches arises from our observation that GWAS/RVAS of mtDNA SNV burden after excluding CH samples reveals a nuclear genetic association landscape that is very similar to that seen for CH itself. Moreover, when we remove these 11,495 individuals, we still see a strong association with hematological malignancies. Our analyses demonstrate that we lack a "gold standard" marker for CH. The development of such a gold standard is required before a true sensitivity/specificity estimate for such a mtSNV count cutoff could be computed.

-Given 50% of CH is 'driverless', and mtDNA SNV burden appears to be replication associated, can the authors identify driverless CH using high age-corrected mtDNA SNVs?

Yes! As stated above, GWAS and RVAS of mtDNA SNV burden with CH individuals excluded revealed a genetic architecture that was virtually unchanged from the same analyses with all individuals included (Figure 3A, EDF10A, Figure 3C, EDF11) implying the identification of so-called "driverless" CH. We have now moved EDF10A to the main figure (new Figure 3B) to highlight this more clearly.

We have now also repeated our correlations between several common diseases and mtDNA SNV burden after exclusion of CH samples and once again we see a highly similar association landscape with exclusion shifting the odds ratio of mtDNA SNV burden on hematologic cancer from 1.29 ($p = 2.33 * 10^{-59}$) to 1.27 ($p = 3.06 * 10^{-47}$).

- Extended Data Figure 2 A/B – mtDNA CN – can the authors comment on the differences in CN between AoU and UKBB cohort? Why does there appear to be greater CN in younger females in UKBB? (Is the same seen in AoU? I could not find these data). Lastly, why does CN reduce with age?

This is an excellent set of questions.

Regarding cross-biobank heterogeneity, as we discuss in Supplementary Note 2 and as we (Gupta et al. 2023 Nature) and others (Fazzini et al. 2018 Sci Rep,) have described in prior work, there are numerous technical variables that can influence measured mtCN. In AoU specifically, we observed a bimodal distribution of mtCN. In this work, we found that mtCN bimodality in AoU was completely explained by DNA extraction kit. As shown in EDF2A, AoU extracted with Autogen has a far more similar mtCN distribution to UKB compared to AoU samples extracted with Chemagen. It is possible that much of the mean difference between AoU Autogen and UKB could be related to technical differences in the extraction processes between the biobanks. We also describe previously that mtCN is correlated with blood cell composition (Gupta et al. 2023 Nature) and given that AoU does not provide blood composition information from the time of blood draw we did not pursue further analysis of mtCN in AoU.

Regarding sex differences in mtCN, while we do not know for sure, it is notable that sex differences appear to vanish on average after the age of 50. This would seem most likely to correlate with menopause in women. In our previous work we found that sex differences (prior to age 50) were even more pronounced prior to blood cell composition correction (Gupta et al. 2023 Nature), implying that there may be some interaction between blood cell composition and menopause as it impacts mtCN.

Regarding the decline in $mtCN_{adj}$ with age -- we do not know the mechanism. We have been interested in investigating this clear decline in $mtCN_{adj}$ with age. It is not explained by a host of technical and biological covariates that we have investigated. To dig into this further, we constructed a new trait – the difference between the predicted age based on $mtCN_{adj}$ and the true chronological age for each individual. Operationally, this involved regressing age onto $mtCN_{adj}$ and then producing the residual. We performed GWAS for this new trait however this analysis produced a few weakly scoring loci that were difficult to interpret. Given the lack of clarity on this result we excluded this from the manuscript. We agree that this is an interesting question one we plan to follow up on.

-Extended Data Fig 12 showing increased mtDNA SNVs (age corrected) in haem cancers is interesting – should this be a main figure, perhaps combining the significant associations into an additional panel in Figure 4?

We have now constructed a smaller version of this figure which has been added as a panel to Figure 4.

- Ordering of results. The section 'Germline variants that predispose to clonal hematopoiesis also predispose to high mtDNA heteroplasmy accrual' – is it worth simply looking at the correlation between CH and mtDNA SNV increase first, prior to a GWAS on increased mtDNA SNV burden? I note this section comes later 'High mtDNA SNV heteroplasmy co-occurs in samples with CH driver mutations', which shows a correlation between rare variants of CH and mtDNA SNV. To me, the first thing to do is to simply find all cases of CH (driver, driverless, copy number mosaic etc) and simply correlate with mtDNA SNV, prior to looking deeper at a GWAS or association with specific variants of CH. I felt this analysis was missing and critical given the conclusion that higher mtDNA SNV reflects hitch-hiking with CH.

For the reasons outlined above – namely, that our results are highly unexpected for the fields of mtDNA biology and aging – we prefer to keep the ordering of our results as they are currently presented.

Referee #2 (Remarks on code availability):

I have not had a chance to review the code for this study.

Referee #3 (Remarks to the Author):

This study leverages the All of Us and UK Biobank datasets to investigate the origins of age-related mtDNA mutations in blood. This work employs numerous approaches to correct the data, enabling this extensive study. The research shows that clonal hematopoiesis (CH) creates an isolating or magnifying effect, revealing low-level mutations. Characterization of these variants suggests that the primary cause of mtDNA mutation with age is deamination in the displaced/heavy strand, with polymerase error being secondary. These mutations do not appear to be under any selection, indicating that they do not affect mitochondrial function. This finding is significant, as I ultimately agree that mtDNA mutations are not a driver of normal aging.

Thank you for your positive remarks.

Even in a clonal expansion condition with nuclear driver variants, these mutations do not reach a high enough burden. Thus, mtDNA mutation accumulation is an aging hallmark.

This is exactly right.

This conclusion would be strengthened if individual cells carrying driver mutations were isolated and sequenced at a single-cell level, but that is merely a nice-to-have and not necessary to support the findings.
Some Points of Novelty:
Several aspects of this study are novel
-Updated mtSwirl (v2) algorithm.
-Largest databases to date
-corrects for mtDNA copy number and distribution of cell types
-corrects for ancestry
-Regresses heteroplasmy to driver nuclear variations and CH. To an extent, this action had to find CH at the core, but that doesn't make it irrelevant or error-prone.
Concerns:
Some issues arise from the manuscript. It was unclear how novel the elements of this study were in the context of the existing literature. Concerns arose when it was observed that the types of age-related mutations had been previously described, and the nuclear mutations driving CH were largely understood. However, I cannot find where the aging mtDNA variants can be highlighted by CH. Driver genes have been well described in CH, but they have not been leveraged to highlight mtDNA mutations. I believe that the aggregate of these finding bear support, especially in light of the number of tools leveraged to correct the data. This work should provide the definitive description of age-related variation and may inspire new studies on mtDNA variation, CH, and aging in disease conditions.

Thank you for your positive remarks. You are correct that the association between age-related mtSNV accrual and CH has not been reported before. This is not something we expected in the mitochondrial field nor anticipated in the aging literature. This has been an unsolved problem in our field and using large scale human genetics we believe we have arrived at a simple and intuitive mechanism explaining much of the observed mtDNA SNV mutation accrual in blood.

Isolated points:
Additional contributions are two new driver genes in CH. It is this reviewer's opinion that this might be more strongly highlighted in the text.

Thank you. We have accordingly added the following to the discussion:

"NEMF and KCNIP3 may represent new putative driver genes; NEMF is a potential tumor suppressor in lung cancer[44] and the D. melanogaster ortholog is thought to regulate the DNA damage response[45]. Larger sample sizes could allow for the discovery of more drivers."

The connection with clonal hematopoiesis and telomere-associated genes is interesting. Given that changes in telomere genes alter clonal hematopoiesis indirectly via telomere length. It would be interesting

to test the association with telomere length and the accumulation of age-associated mitochondria variants.

Thank you for this excellent idea, Reviewer #2 also made this suggestion. We have upgraded our revision with an analysis of the relationship between germline variants in TERT, telomere length, mtSNV burden, and mtCNadj. We have included the following new text:

"Given the rich literature linking telomerase and aging, we next evaluated for evidence of colocalization of GWAS signal in the *TERT* locus for mtCN (Extended Data Figure 2C) and mtDNA SNV burden (Figure 3A). The variant rs7705526 (chr5:1285859:C:A) had a posterior inclusion probability (via fine-mapping) of ~1 for mtDNA SNV burden and 0.39 for mtCN suggesting co-localization (colocalization posterior probability, CLPP = ~0.39). This association also colocalizes with a recent GWAS of clonal hematopoiesis[28] (CLPP = 1) and a recent GWAS of telomere length[31] (CLPP = 1). Thus, based on our current work and prior studies, this *TERT* variant: (i) increases $mtCN_{adj}$, (ii) increases mtDNA SNV burden, (iii) increases CH risk, and (iv) increases telomere length.

We obtained telomere lengths from UKB and explored their correlation with mtDNA phenotypes (Methods). After correction for covariates including age, we observed a strong positive correlation between $mtCN_{adj}$ and telomere length (Extended Data Figure 15A). In contrast, mtSNV burden did not correlate with telomere length. This is not entirely unexpected – recent work has shown that genetic predisposition to longer telomere length actually associates with higher CH, while CH subsequently results in a decline in telomere length[32]. In agreement, our fine mapping indicates that the likely causal variant at *TERT* increases telomere length and CH risk. This same variant increases mtSNV count. We speculate that a genetic predisposition for longer telomeres may increase mtSNV count by increasing CH predisposition, however this effect is masked by the fact that CH itself results in more cell replications which reduces telomere length but increases heteroplasmic mtSNV count."

While it is likely out of the scope of the current manuscript, it would be an interesting comparison to examine the mitochondrial variants in two tissues, one hematopoietic and one non-hematopoietic, from the same individual to determine if the same variants are present. This experiment would inform when the variants likely arose.

We agree that this would be extremely interesting, but it is outside the scope of the current paper. We end our paper by remarking that future studies will be required to decipher the cause of age associated mtDNA mutation accrual in solid organs.

The literature is in dire need of a reset on the role of oxidative stress-driven mutations in aging. The authors are invited to strengthen this issue in the manuscript and reduce other redundancies in the discussion.

Thank you. We have attempted to emphasize the classical, ROS-based model in the introduction to motivate our study:

"The mechanism of this age accrual of heteroplasmic mtSNVs in blood is unknown. Classically, oxidative damage to mtDNA from reactive oxygen species was invoked as a part of a "vicious cycle" where mtDNA mutations lead to further reactive oxygen species and more mutations[3–5]. More recent studies of aging brains[6] and tumor samples[7] have questioned the role of oxidative damage[8] in mutation generation. Once individual mutations do arise, it is unclear how they become abundant enough to detect. Here, we investigate why mtSNVs accumulate with age in blood."

CH could significantly influence the variable presentation of phenotypes in mtDNA disease patients. This concept may deserve a substantial comment from the authors in the discussion.

Thank you for this interesting idea. Referee #1 also asked us to comment on how findings from the current work may impact the rare mitochondrial disorders field. We now comment:

"An interesting possibility arising from our work is that in some instances of mtDNA disease, high levels of mutant heteroplasmy may be maintained due nuclear genomic driver mutations. Joint sequencing of nuclear and mtDNA genomes in patients with maternally transmitted or sporadic mtDNA mutations could be highly informative."

Minor comments for the authors:
- This manuscript is generally well written. I suggest reducing redundancy between results and discussion and incorporating some emphatic language to emphasize the novelty and significance of the findings. The manuscript feels understated.

Thank you for your encouragement. We have revised the text to emphasize more the novelty and significance.

-Sentence construction using this or these as the lone subject is prone to confusion. Please augment these sentences with a noun for better connectivity. Examples include line 187, but this is not comprehensive

We have addressed these instances for added clarity.

-I believe the word "strand" is missing from line 199.

Fixed, thank you.

-In general, the manuscript is written to a mitochondrially-focused

audience and may not be accessible to the broad readership of Nature. For example, most readers may not be familiar with references to the heavy strand in the mitochondrial genome or OL. Some context for these concepts would improve the readability.

We have clarified these concepts in the main text so they are more accessible to a general audience.

-The authors should be careful with terminology around "mutations" and "variants". In general, "variant" is preferred. If both are used, "mutation" should refer to changes that have known negative effects. For example, the authors use the term "TERT mutation" to refer to variants (SNPs) that are found in and around the TERT gene and are associated with specific phenotypes. For the majority of these variants, no functional studies have been performed to understand their role and it is unclear if they are functional or in disequilibrium with the functional variant.

We now refer to our nuclear GWAS loci (e.g., *TERT*) as "variants," but when we talk about the mtSNV accrual, we refer to those as mutations that are accruing with aging. These are likely replication errors arising from a known mutational process.

Referee #3 (Remarks on code availability):

I was unable to review the code. This should be done via specialist review.

Referee #4 (Remarks to the Author):

I co-reviewed this manuscript with one of the reviewers who provided the listed reports.

Editorial comments:

1. Please reduce the length of the title to 75 characters (with spaces) or fewer, so that it fits on two lines in the final layout.

We have shortened the title to 71 characters with spaces:

"Mechanism of age-related accumulation of mtDNA mutations in human blood"

2. Please provide the manuscript in .docx format. Currently it is in pdf format.

Done.

3. Please add references to the abstract.

Done.

4. Please split the main text and methods references into two separate, but continuously numbered, lists that follow the discussion and the methods section, respectively.

Done.

5. The Chief Editor has approved 4,800 words and 5 main display items for your paper. As you are aware, the current manuscript exceeds this limit by quite some margin. We ask that you make the necessary revisions to bring the article to budget. Please turn one of the main figures into an Extended Data (ED) Figure (I recommend this to be Figure 6; main figures should generally show bona fide research results while schematic overviews should be Extended Data figures). We further recommend that you aim for more economical writing and to move more technical and/or peripheral descriptions and discussions of results to Supplementary Notes.

We have now truncated the text. The text (including the introduction) is now 4785 words. As discussed, we have substantially reduced the size of figure 5 and retained a smaller version of figure 6. We have also greatly reduced the size of figure 1.

6. Please remove the main figures from the article file and re-supply them individually in an acceptable format such as EPS, AI, PS, PDF, PPT, PSD or XLS (for graphs) with editable vector files.

We have now removed main figures from the article file.

7. Please reduce subheadings to 40 characters (with spaces) or fewer.

We have now greatly shortened all subheadings.

8. You have more than one account on EJP. Please contact Nature Manuscripts (naturemanuscripts@nature.com) to confirm all accounts which belong to you, and your primary email address.

Done.

9. Please provide a supplementary information guide as a separate word document.

Done.

10. We can allow up to 12 ED Figures. Please consolidate the current ED Figures into 12 by compiling multi-panel figures (each can be up to an entire A4 page in size). If necessary, you may move some ED Figures or parts of them to the Supplementary Information.

Done.

11. Please make sure to provide a third party rights table (more information below) when you resubmit. If Biorender or similar software has been used, please also ensure to provide relevant licenses.

We have no figures that use third party assets. We have included a completed rights table anyway.

12. Please remove the Extended data figures from the article file and re-supply them individually in EPS, JPEG or TIF format.

Done.

13. Please remove the supplementary information from the article file and resupply it as separate file.

Done.

14. Please ensure that the text size in all figures is at least 5 pt Arial, keeping in mind that main figures cannot exceed 18cm in width and 17cm in height.

Confirmed!

15. Please deposit and make available all data (i.e. they should be live) before the final resubmission of the manuscript.

This is underway. We have made all meta-analyses available in the AllofUs researcher workbench under the workspace titled “Mechanism of age-related accumulation of mtDNA mutations in human blood”. This workspace also contains raw calls from AoU. We are in the process of returning UKB data to the UK Biobank for dissemination.

16. For any Supplementary Figures, please check and confirm that:
* If data is presented as bar charts, individual data points are shown using overlaid dot plots.
* The n number (i.e. the sample size used to derive statistics) is provided and defined as a precise value (not a range), using the wording “n=X samples/cells/independent experiments” etc. where applicable.
* Any chart axis, error bars, scale bars, symbols and colour scales are defined.
* Any statistical tests used for data analysis are specified and exact p-values are provided either on the figures themselves, in the legend or in the Source Data file.

* Wherever representative data such as micrographs are shown, the legend indicates how many times the experiment was repeated with the same results.

Done.

Referee comments:

Referee #1 (Remarks to the Author):

The authors have addressed my comments appropriately.
Guillaume Lettre

Thank you for your input and feedback.

Referee #2 (Remarks to the Author):

The concept that passenger mitochondrial mutations are detectable in blood due to them hitchhiking with clonal haematopoiesis seems expected but has not been confirmed at population level before. This manuscript carefully shows that this is indeed the case and that such mitochondrial mutations accrue due to replication errors. The authors show that there is no evidence of mitochondrial 'hypermutation' driven by germline nuclear variants, and that there is an absence of specific mitochondrial mutations driving positive selection in blood over age. These results are important and clarify confusion in the field around any causal contribution of mitochondrial mutations to mutational landscapes and clonal architecture in blood. The dataset is the largest to date, with validation using independent datasets, and will be relevant to several research communities.

Thank you for addressing my queries. I have no further comments.

Thank you for your input and feedback.

Referee #3 (Remarks to the Author):

The authors were responsive to the original positive review.

I have minor points for the authors:
1) mtCN is never defined in the manuscript.

Thank you for catching this – this is now defined.

2) The text “Given the rich literature linking telomerase and aging…” would be more accurately stated “Given the rich literature linking telomere length and aging…”. Telomere length shortens with age.

We have now adjusted this to “Given the rich literature linking the TERT gene product telomerase with telomere length and aging…”.

3) Regarding mutation and variant, there may be a tendency to see "mutation" as impactful and "variant" as unimpactful. I would suggest that age-acquired mutations be initially described as neutral mutations to prevent this erroneous connection for general readers.

Thank you for this input. We have now updated the abstract to state: “We observe that mtDNA single nucleotide variants (mtSNVs) accumulate sharply at age 60, occur at low levels of heteroplasmy, and exhibit little evidence of positive selection and are likely predominantly neutral.” In the main text, we have re-titled the third section of the results to state “Age-accumulating mtSNVs appear neutral” which should make this clear for the reader.

Referee #4 (Remarks to the Author):

I co-reviewed this manuscript with one of the reviewers who provided the listed reports.

Thank you!
